# Medical and neurobehavioural phenotypes in male and female carriers of Xp22.31 duplications in the UK Biobank

**DOI:** 10.1093/hmg/ddaa174

**Published:** 2020-08-07

**Authors:** Samuel J A Gubb, Lucija Brcic, Jack F G Underwood, Kimberley M Kendall, Xavier Caseras, George Kirov, William Davies

**Affiliations:** Division of Psychological Medicine and Clinical Neurosciences and Centre for Neuropsychiatric Genetics and Genomics, School of Medicine, Cardiff University, Cardiff CF24 4HQ, United Kingdom; School of Psychology, Cardiff University, Cardiff CF10 3AT, United Kingdom; Division of Psychological Medicine and Clinical Neurosciences and Centre for Neuropsychiatric Genetics and Genomics, School of Medicine, Cardiff University, Cardiff CF24 4HQ, United Kingdom; Neuroscience and Mental Health Research Institute, Cardiff University, Cardiff CF24 4HQ, United Kingdom; Division of Psychological Medicine and Clinical Neurosciences and Centre for Neuropsychiatric Genetics and Genomics, School of Medicine, Cardiff University, Cardiff CF24 4HQ, United Kingdom; Division of Psychological Medicine and Clinical Neurosciences and Centre for Neuropsychiatric Genetics and Genomics, School of Medicine, Cardiff University, Cardiff CF24 4HQ, United Kingdom; Division of Psychological Medicine and Clinical Neurosciences and Centre for Neuropsychiatric Genetics and Genomics, School of Medicine, Cardiff University, Cardiff CF24 4HQ, United Kingdom; Division of Psychological Medicine and Clinical Neurosciences and Centre for Neuropsychiatric Genetics and Genomics, School of Medicine, Cardiff University, Cardiff CF24 4HQ, United Kingdom; School of Psychology, Cardiff University, Cardiff CF10 3AT, United Kingdom; Neuroscience and Mental Health Research Institute, Cardiff University, Cardiff CF24 4HQ, United Kingdom

## Abstract

Deletions spanning the *STS* (steroid sulfatase) gene at Xp22.31 are associated with X-linked ichthyosis, corneal opacities, testicular maldescent, cardiac arrhythmia, and higher rates of developmental and mood disorders/traits, possibly related to the smaller volume of some basal ganglia structures. The consequences of duplication of the same genomic region have not been systematically assessed in large or adult samples, although evidence from case reports/series has indicated high rates of developmental phenotypes. We compared multiple measures of physical and mental health, cognition and neuroanatomy in male (n = 414) and female (n = 938) carriers of 0.8–2.5 Mb duplications spanning *STS*, and non-carrier male (n = 192, 826) and female (n = 227, 235) controls from the UK Biobank (recruited aged 40–69 from the UK general population). Clinical and self-reported diagnoses indicated a higher prevalence of inguinal hernia and mania/bipolar disorder respectively in male duplication carriers, and a higher prevalence of gastro-oesophageal reflux disease and blistering/desquamating skin disorder respectively in female duplication carriers; duplication carriers also exhibited reductions in several depression-related measures, and greater happiness. Cognitive function and academic achievement did not differ between comparison groups. Neuroanatomical analysis suggested greater lateral ventricle and putamen volume in duplication carriers. In conclusion, Xp22.31 duplications appear largely benign, but could slightly increase the likelihood of specific phenotypes (although results were only nominally-significant). In contrast to deletions, duplications might protect against depressive symptoms, possibly via higher *STS* expression/activity (resulting in elevated endogenous free steroid levels), and through contributing towards an enlarged putamen volume. These results should enable better genetic counselling of individuals with Xp22.31 microduplications.

## Introduction

The X-linked *STS* gene encodes the enzyme steroid sulfatase which catalyses the desulfation of sulfated steroids (e.g. dehydroepiandrosterone sulfate, DHEAS) to their free steroid counterparts; these may subsequently act as precursors for a variety of androgens and oestrogens (1). Genetic deletions at Xp22.31 encompassing *STS* are associated with the rare skin condition X-linked ichthyosis (XLI, OMIM: 308100) (2). XLI predominantly affects males, and is associated with a number of conditions including cryptorchidism and benign corneal opacities; some female deletion carriers also have corneal opacities and can exhibit prolonged/delayed labour during childbirth as a consequence of STS deficiency in the fetal portion of the placenta (2). We have recently shown that males with deletions spanning *STS* are at significantly increased risk of atrial fibrillation/flutter compared to male non-carriers (3). Occasional comorbidities with XLI can include focal epilepsy (4), pyloric hypertrophy, congenital defect of the abdominal wall, acute lymphoblastic leukaemia, bilateral periventricular nodular heterotopia and end-stage renal failure (2). Deletions around *STS* are also associated with substantially elevated rates of developmental disorders (notably autism and inattentive Attention Deficit Hyperactivity Disorder (ADHD)) (5–7), and with elevated rates of associated cognitive, motor and mood symptoms (3, 8, 9); these psychological features may stem from subcortical brain structure differences, notably a reduction in the size of some regions of the basal ganglia (3). Rare, larger genetic deletions around *STS* are associated with a number of more severe neurological conditions including Kallman, Rud, and Conradi syndromes (2).

Whilst the phenotypes associated with XLI-associated deletions are reasonably well-characterised, the phenotypes associated with the reciprocal duplications have yet to be investigated systematically in large or adult samples. Case series/studies documenting predominantly young, clinically-ascertained, individuals with Xp22.31 duplications report a number of neurological features including intellectual disability/cognitive impairment (40%), autism or autistic behaviours (10–20%), global developmental delay (10%), delayed speech and language (10%) and seizures (10%), as well as other less common clinical manifestations including microcephaly (5–10%), muscular hypotonia (5–10%) and clinodactyly or shortness of the fifth finger (5%) (10–20), and (Supplementary Material, Table 1). Whether the Xp22.31 duplication is pathogenic (particularly with regard to its role in brain function and head/facial dysmorphism), or whether it is benign but tends to occur in combination with other pathogenic mechanisms, has been a matter for debate (21–23).

In this study, we exploited the power of the genotyped and extensively-characterised large UK Biobank sample comprising adults recruited from the general population of the UK (24) to compare a wide range of physical and psychiatric illnesses (and treatments), cognitive function, and subcortical brain structure, in male and female Xp22.31 duplication carriers to that of sex-matched control subjects not carrying the variant of interest. Triangulation of multiple objective and self-report Biobank measures is likely to provide comprehensive and accurate information about an individual’s phenotype, including their mental health (25). *A priori*, we considered the possibilities that duplication carriers within our sample may exhibit similar, contrasting, and/or distinct phenotypes to deletion carriers, or that duplications carriers may exhibit few, if any, phenotypic differences from non-carrier controls.

## Results

### Identification and characterisation of duplication cases

We identified 414 male and 938 female duplication carriers, alongside 192 826 male and 227 235 female non-carriers, giving prevalence rates of $\sim$1/470 (0.21%) in males and $\sim$1/240 (0.41%) in females. The mean duplication size was 1.60 ± 0.46 Mb from ChrX:6477469-8 080 874 encompassing the *HDHD1*/*PUDP* (OMIM:306480), *STS* (OMIM:300747), *VCX* (OMIM:300229) and *PNPLA4* (OMIM:300102) genes and the *MIR4767* microRNA.

### ICD-10 diagnoses

We identified ICD-10 unique descriptive codes that were the most common in duplication carriers (present in at least 1 in 40 (>2.5%) carriers), and that were also recorded in a significant proportion (>1.5%) of individuals in the overall male and female samples; these selection criteria, analogous to those used in our previous Xp22.31 deletion study (3), were designed to detect robust between-group effects. 13 ICD-10 codes met these criteria in the male sample (Supplementary Material, Table 2). Of these, just one ‘Unilateral or unspecified inguinal hernia, without obstruction or gangrene’ was nominally-significantly more commonly observed in male duplication carriers than in male non-carrier controls (9.4% vs. 6.8%, OR: 1.42 (95% CI: 1.02–1.97), χ^2^[1] = 3.95, *P* = 0.047); this result did not remain significant after correction for multiple testing. We have previously shown that male deletion carriers in the UK Biobank exhibit significantly increased prevalence of ‘atrial fibrillation/flutter’ and ‘skin of other and unspecified parts of face’ relative to male non-carrier controls; analysis of these two phenotypes in male duplication carriers revealed no significant difference from non-carrier controls (atrial fibrillation: 2.4% vs. 2.7% respectively, OR: 0.88 (95% CI: 0.47–1.66), χ^2^[1] = 0.06, *P* = 0.82; Skin: 1.7% vs. 1.5% respectively, OR: 1.12 (95% CI: 0.53–2.37), χ^2^[1] = 0.009, *P* = 0.92).

We identified 16 ICD-10 unique descriptive codes that met the aforementioned selection criteria in the female sample (Supplementary Material, Table 3). Of these, only one, ‘Gastro-oesophageal reflux disease (GORD) without oesophagitis’ was significantly more common in duplication carriers than in non-carrier controls (2.7% vs. 1.6%, OR: 1.69 (95% CI: 1.13–2.51), χ^2^[1] = 4.88, *P* = 0.027); again, this finding did not survive correction for multiple testing.

The number of ‘inguinal hernia’ diagnoses did not differ significantly between female duplication carriers and female non-carrier controls (0.6% vs. 0.4% respectively, OR: 1.62 (95%CI: 0.72–3.63), *P* = 0.20), nor did the number of ‘GORD without oesophagitis’ diagnoses differ between male duplication carriers and male non-carrier controls (1.0% vs. 1.2% respectively, OR: 0.82 (95%CI: 0.31–2.19), *P* = 1.00).

Clinical diagnosis rates of developmental and mood/anxiety disorders were not significantly different between male and female duplication and sex-matched control cohorts (Supplementary Material, Tables 4 and 5), although baseline diagnosis rates were low across all groups.

### Self-reported non-cancer illnesses

We analysed a number of medical and psychiatric/neurological self-reported diagnoses across all groups; the phenotypes we assessed were indicated by the ICD-10 data above, were related to tissues with high *STS* expression, or were potentially sensitive to dosage of the region of interest as indicated by previous deletion carrier data.

No physical health conditions we assessed were self-reported at significantly higher rates in male duplication carriers compared to male non-carrier controls, although rates of ‘inguinal hernia’ and ‘gastro-oesophageal reflux’ tended to be higher in the former than the latter group (1.9% vs. 0.9%, *P* = 0.065; 6.0% vs. 4.7%, χ^2^[1] = 1.44, *P* = 0.20 respectively) (Supplementary Material, Table 6). Only one self-reported psychiatric condition (‘mania/bipolar disorder’) differed in prevalence between male groups with higher rates in the duplication group compared to the control group (1.0% vs. 0.3%, *P* = 0.026); this finding did not survive correction for multiple testing (corrected *P*-value>0.99) (Supplementary Material, Table 6).

Only one of the physical health conditions we assessed (‘blistering/desquamating skin disorder’) was significantly more commonly self-reported in female duplication carriers compared to female non-carrier controls (0.5% vs. 0.1%, *P* = 0.013), although this result did not withstand multiple testing correction (corrected *P* = 0.69); the self-reported prevalence of ‘inguinal hernia’ and ‘gastro-oesophageal reflux’ was similar in female duplication carriers and in female controls (0.1% vs. 0.1%, *P* = 0.51; 4.1% vs. 4.4%, χ^2^[1] = 0.20, *P* = 0.63) (Supplementary Material, Table 7). No psychiatric/neurological conditions we assessed differed in self-reported prevalence between female duplication and control groups (Supplementary Material, Table 7).

### Mental Health Questionnaire (MHQ)

The MHQ was completed by a total of 129 male duplication carriers, 59 418 male controls, 269 female duplication carriers and 76 265 female controls. Neither male nor female duplication carriers completing the MHQ self-reported differing frequencies of mental distress or mental health diagnoses relative to sex-matched controls (Table 1).

**Table 1:** Frequency of mental distress, help-seeking behaviour and psychiatric illness in male and female duplication carriers and sex-matched controls completing the Mental Health Questionnaire

Measure	Male control	Male duplication	Statistical analysis	Female control	Female duplication	Statistical analysis
Mental distress preventing usual activities (Yes/No)	15 229/43273	34/94	χ^2^_[1]_ = 0.001, *P* = 0.971	29 180/46221	100/165	χ^2^_[1]_ = 0.067, *P* = 0.796
Or ever sought/received help for mental distress (Yes/No)	17 695/41526	35/94	χ^2^_[1]_ = 0.342, *P* = 0.559	35 046/40939	115/152	χ^2^_[1]_ = 0.878, *P* = 0.349
Social anxiety or social phobia (Yes/No)	771/58647	1/128	*P* > 0.99	888/75377	5/264	*P* = 0.250
Phobia (other than social or agoraphobia) (Yes/No)	586/58832	1/128	*P* > 0.99	1256/75009	2/267	*P* = 0.337
Panic attacks (Yes/No)	2296/57122	8/121	*P* = 0.166	5189/71076	14/255	χ^2^_[1]_ = 0.845, *P* = 0.358
Obsessive compulsive disorder (Yes/No)	336/59082	2/127	*P* = 0.167	507/75758	1/268	*P* > 0.99
Mania/Bipolar (Yes/No)	309/59109	0/129	*P* > 0.99	371/75894	2/267	*P* = 0.378
Depression (Yes/No)	9295/50123	16/113	χ^2^_[1]_ = 0.794, *P* = 0.373	19 554/56711	57/212	χ^2^_[1]_ = 2.557, *P* = 0.110
Bulimia (Yes/No)	18/59400	0/129	*P* > 0.99	388/75877	1/268	*P* > 0.99
Anxiety, nerves or generalised anxiety disorder (Yes/No)	6369/53049	13/116	χ^2^_[1]_ = 0.009, *P* = 0.926	12 870/63395	35/234	χ^2^_[1]_ = 2.586, *P* = 0.108
Anorexia (Yes/No)	30/59388	0/129	*P* > 0.99	721/75544	3/266	*P* = 0.745
Agoraphobia (Yes/No)	119/59299	0/129	*P* > 0.99	413/75852	1/268	*P* > 0.99
ADHD (Yes/No)	50/59368	1/128	*P* = 0.105	46/76219	0/269	*P* > 0.99

Male duplication carriers differed from sex-matched controls most frequently with respect to depression-related symptoms and treatments (Supplementary Material, Table 8). Specifically, compared to controls, duplication carriers reported a lower prevalence of ‘prolonged feelings of sadness or depression’ (34% vs. 44%, OR: 0.66 (95%CI: 0.46–0.96), χ^2^[1] = 4.51, *P* = 0.034), reduced ‘recent feelings of tiredness or low energy’ (*P* = 0.022, d < 0.02), lower ‘number of lifetime depressed periods’ (*P* = 0.049, d < 0.03) and elevated levels of ‘general happiness’ (*P* = 0.019, d < 0.02); those male duplication carriers who did experience depressive episodes, less frequently experienced ‘feelings of worthlessness during the worst period of depression’ (32% vs. 48%, OR: 0.50 (95%CI:0.26–0.94), χ^2^[1] = 4.16, *P* = 0.041) and were more likely to engage in talking therapies (38% vs. 15%, OR:3.36 (95%CI:1.59–7.11), χ^2^[1] = 4.16, *P* = 0.041). These effects in the male duplication carrier group are unlikely to be a consequence of lower exposure to traumatic events, in that this group generally reported experiencing similar levels of adverse events to the control group, and actually experienced a diagnosis of a life-threatening illness significantly more commonly (*P* = 0.049, d < 0.015) (Supplementary Material, Table 8). Male duplication carriers did not self-report differently to non-carrier controls with respect to manic/bipolar disorder symptoms, anxiety-related symptoms, addictive behaviours, alcohol or cannabis use, or unusual and psychotic experiences (Supplementary Material, Table 8).

Female duplication carriers also differed from sex-matched controls most consistently with respect to depression-related symptoms and treatments (Supplementary Material, Table 9). Fewer duplication carriers reported sleep changes during depressive episodes (76% vs. 83%, OR:0.62 (95%CI:0.42–0.91), χ^2^[1] = 5.50, *P* = 0.019) and fewer reported ‘difficulty concentrating during their worst depressive episode’ (72% vs. 81%, OR:0.61 (95%CI: 0.42–0.87), χ^2^[1] = 6.89, *P* = 0.009). Female duplication carriers were also less likely to have taken prescribed substances for depression than female controls (22% vs. 30%, OR:0.65 (95%CI:0.49–0.87), χ^2^[1] = 7.96, *P* = 0.005), but more likely to have taken alcohol/drugs to manage depressive symptoms (10% vs. 7%, OR:1.62 (95%CI:1.09–2.40), χ^2^[1] = 5.32, *P* = 0.021). A smaller proportion of female duplication carriers than controls who experienced anxiety self-reported being easily tired during their worst episode (65% vs. 76%, OR: 0.61 (95%CI:0.39–0.96), χ^2^[1] = 4.02, *P* = 0.045), but a significantly larger proportion of duplication carriers reported using alcohol/drugs to manage anxiety symptoms (8% vs. 5%, OR:1.63 (95%CI:1.05–2.53), χ^2^[1] = 4.33, *P* = 0.037). Duplications in female participants were not associated with manic/bipolar disorder symptoms, addictive behaviours, alcohol or cannabis use, unusual and psychotic experiences, experiencing traumatic events, or happiness/subjective wellbeing (Supplementary Material, Table 9).

### Medications

We compared prescription rates of medications for gastric reflux, blistering/desquamating disorders and psychiatric conditions indicated above in male and female duplication carriers and controls. There was a trend for male duplication carriers to be prescribed proton pump inhibitors more frequently than male controls (11.1% vs. 8.3%, OR:1.38 (95%CI:1.01–1.87), χ^2^[1] = 3.85, *P* = 0.050, corrected *P* = 0.40), but there was no between-group difference for antacids or H2 receptor antagonists; prescription rates of psychiatric medications were similar between male duplication carriers and male controls (Supplementary Material, Table 10). Female duplication carriers and controls were prescribed all of the aforementioned medications at equivalent rates (Supplementary Material, Table 11).

### Academic qualifications and cognitive function

Male and female duplication carriers did not differ significantly from their sex-matched controls in terms of their highest academic qualification (Table 2). Regression analysis controlling for age at testing indicated that male duplication carriers performed equivalently to male controls across all of the cognitive measures we assessed (Table 3). Female duplication carriers performed similarly to female controls on all measures apart from the number of correct substitutions within the Symbol Digit Substitution task (an index of complex processing), where they performed slightly worse (B = -0.254 ± 0.108, β = −0.019, *P* = 0.019); this result did not remain significant after correction for multiple testing (Table 3).

**Table 2:** Highest academic qualification achieved by male and female duplication carriers and sex-matched controls

Highest academic qualification	Male control (n = 182 574)	Male duplication (n = 396)	Statistical analysis	Female control (n = 212 235)	Female duplication (n = 871)	Statistical analysis
College/University degree	62 833 (34%)	140 (35%)	χ^2^_[1]_ = 8.117, *P* = 0.149	67 597 (32%)	285 (33%)	χ^2^_[1]_ = 2.097, *P* = 0.836
A/AS Levels	20 020 (11%)	34 (9%)		27 150 (13%)	116 (13%)	
O Levels/GCSEs	36 866 (20%)	73 (18%)		55 048 (26%)	208 (24%)	
CSEs	10 595 (6%)	34 (9%)		12 524 (6%)	50 (6%)	
NVQ/HND/HNC	17 905 (10%)	41 (10%)		10 069 (5%)	43 (5%)	
None	33 959 (19%)	74 (19%)		39 847 (19%)	169 (19%)	

**Table 3:** Performance on key measures of seven cognitive tasks by male and female duplication carriers and sex-matched controls

Cognitive task	Control group	Duplication group	Statistical analysis	Benjamini-Hochberg corrected *P*-value
*Male participants*				
Pairs Matching Test (total number of errors)	−0.048 (−0.038- -0.058) (n = 43 575)	0.058 (−0.123–0.239) (n = 103)	B = 0.106 ± 0.102, β = 0.005, *P* = 0.297	0.520
Reaction Time Test	−0.087 (−0.078- -0.096) (n = 43 203)	0.010 (−0.175–0.195) (n = 101)	B = 0.098 ± 0.093, β = 0.005, *P* = 0.295	0.520
Fluid Intelligence Test (total number of correct answers)	0.162 (0.180–0.144) (n = 13 251)	−0.030 (−0.397–0.336) (n = 34)	B = -0.192 ± 0.181, β = −0.009, *P* = 0.288	0.520
Digit Span Test (maximum number of digits remembered)	0.147 (0.118–0.176) (n = 4242)	0.5031 (−0.040–1.047) (n = 13)	B = 0.362 ± 0.267, β = 0.021, *P* = 0.176	0.520
Symbol Digit Substitution Test (number of correct substitutions)	−0.010 (−0.026–0.005) (n = 11 768)	−0.030 (−0.295–0.234) (n = 30)	B = -0.022 ± 0.158, β = −0.001, *P* = 0.887	0.887
Trail Making Test A (Time to completion)	−0.059 (−0.0760- -0.042) (n = 10 669)	−0.011 (−.0.326- -0.305) (n = 30)	B = 0.052 ± 0.165, β = 0.003, *P* = 0.751	0.876
Trail Making Test B (Time to completion)	−0.043 (−0.026- -0.060) (n = 10 677)	0.007 (−0.363–0.378) (n = 30)	B = 0.055 ± 0.164, β = 0.003, *P* = 0.736	0.876
*Female participants*				
Pairs Matching Test (total number of errors)	−0.008 (−0.017–0.000) (n = 54 232)	0.074 (−0.061–0.209) (n = 231)	B = 0.084 ± 0.066, β = 0.005, *P* = 0.206	0.288
Reaction Time Test	0.102 (0.095–0.110) (n = 53 772)	0.128 (0.003–0.252) (n = 229)	B = 0.030 ± 0.060, β = 0.002, *P* = 0.620	0.620
Fluid Intelligence Test (total number of correct answers)	0.046 (0.031–0.061) (n = 16 651)	−0.030(−0.289–0.230) (n = 67)	B = -0.076 ± 0.119, β = −0.005, *P* = 0.526	0.614
Digit Span Test (maximum number of digits remembered)	−0.034 (−0.060- -0.008) (n = 5404)	0.338 (−0.112–0.789) (n = 18)	B = 0.372 ± 0.226, β = 0.022, *P* = 0.100	0.175
Symbol Digit Substitution Test (number of correct substitutions)	−0.026 (−0.040- -0.011) (n = 15 156)	−0.277 (−0.504- -0.050) (n = 71)	B = -0.254 ± 0.108, β = −0.019, *P* = 0.019	0.133
Trail Making Test A (Time to completion)	0.101 (0.084–0.117) (n = 13 173)	0.303 90.072–0.535) (n = 64)	B = 0.203 ± 0.120, β = 0.015, *P* = 0.090	0.175
Trail Making Test B (Time to completion)	0.078 (0.063–0.094) (n = 13 200)	0.271 (0.051–0.490) (n = 64)	B = 0.193 ± 0.114, β = 0.015, *P* = 0.091	0.175

### Intra-cranial volume and subcortical neuroanatomy

As both microcephaly and macrocephaly have been reported in duplication carriers, we initially tested whether intra-cranial volume (a surrogate measure of skull size) was associated with duplication carrier status after adjusting for age at scanning, handedness and scanning centre; this did not appear to be the case for either male (B = 10.7 ± 53.4, β = 0.004, *P* = 0.84) or female (B = 15.5 ± 27.7, β = 0.010, *P* = 0.58) participants. Regression analysis adjusting for age at scanning, intra-cranial volume, handedness and scanning centre, indicated a nominally-significantly enlarged left lateral ventricle size in male duplication carriers compared to controls (B = 4.58 ± 2.20, β = 0.036, *P* = 0.038), a result which did not survive correction for multiple testing; however, consistent with this, male duplication carriers exhibited a trend towards enlarged right lateral ventricle volumes, and female duplication carriers also exhibited enlarged left and right lateral ventricles on average relative to female controls (Table 4). Equivalent regression analysis in females indicated a nominally-significantly enlarged left putamen in duplication carriers relative to controls (B = 0.31 ± 0.14, β = 0.037, *P* = 0.033, corrected *P* = 0.53); both left and right putamen volumes were, on average, also higher in male duplication carriers than male controls (Table 4).

**Table 4:** Volumes of eight subcortical brain regions (right and left hemisphere) in male and female duplication carriers and sex-matched controls

Subcortical region	Control group (cm^3^)	Duplication group (cm^3^)	Statistical analysis	Benjamini-Hochberg corrected *P*-value
**Male participants**				
*Left hemisphere*				
Lateral ventricle	13.86 (13.72–14.00) (n = 8268)	17.11 (13.78–20.45) (n = 20)	B = 4.58 ± 2.20, β = 0.036, *P* = 0.038	0.56
Thalamus	7.98 (7.96–8.01) (n = 8536)	7.38 (6.81–7.94) (n = 20)	B = -0.45 ± 0.36, β = −0.020, *P* = 0.202	0.85
Caudate	3.58 (3.57–3.59) (n = 8508)	3.51 (3.36–3.66) (n = 20)	B = -0.077 ± 0.16, β = −0.008, *P* = 0.629	0.98
Putamen	5.27 (5.25–5.28) (n = 8550)	5.38 (5.13–5.63) (n = 20)	B = 0.011 ± 0.27, β = 0.001, *P* = 0.968	0.98
Pallidum	1.37 (1.36–1.37) (n = 8544)	1.32 (1.30–1.42) (n = 20)	B = 0.028 ± 0.097, β = 0.006, *P* = 0.772	0.98
Hippocampus	4.31 (4.30–4.32) (n = 8539)	4.25 (4.03–4.46) (n = 20)	B = -0.015 ± 0.17, β = −0.002, *P* = 0.929	0.98
Amygdala	1.63 (1.63–1.64) (n = 8531)	1.62 (1.52–1.72) (n = 19)	B = -0.073 ± 0.086, β = −0.015, *P* = 0.398	0.98
Nucleus accumbens	5.28 (5.26–5.31) (n = 8547)	5.54 (4.86–6.22) (n = 20)	B = 0.011 ± 0.045, β = 0.004, *P* = 0.806	0.98
*Right Hemisphere*				
Lateral ventricle	12.88 (12.75–13.01) (n = 8279)	14.93 (12.12–17.67) (n = 20)	B = 2.55 ± 2.05, β = 0.021, *P* = 0.213	0.85
Thalamus	6.93 (6.92–6.95) (n = 8518)	6.71 (6.38–7.04) (n = 20)	B = -0.0065 ± 0.23, β = 0.000, *P* = 0.977	0.98
Caudate	3.75 (3.74–3.76) (n = 8512)	3.74 (3.56–3.92) (n = 20)	B = -0.075 ± 0.17, β = −0.008, *P* = 0.651	0.98
Putamen	5.05 (5.04–5.06) (n = 8540)	5.14 (4.89–5.40) (n = 20)	B = -0.010 ± 0.22, β = −0.001, *P* = 0.963	0.98
Pallidum	1.58 (1.58–1.59) (n = 8517)	1.62 (1.55–1.70) (n = 20)	B = 0.054 ± 0.076, β = 0.013, *P* = 0.480	0.98
Hippocampus	4.46 (4.45–4.47) (n = 8535)	4.37 (4.14–4.56) (n = 20)	B = 0.029 ± 0.17, β = 0.003, *P* = 0.863	0.98
Amygdala	1.70 (1.70–1.71) (n = 8538)	1.68 (1.55–1.81) (n = 19)	B = -0.016 ± 0.087, β = −0.003, *P* = 0.858	0.98
Nucleus accumbens	5.68 (5.66–5.70) (n = 8546)	6.06 (5.60–6.52) (n = 20)	B = 0.073 ± 0.040, β = 0.033, *P* = 0.070	0.56
**Female participants**				
*Left hemisphere*				
Lateral ventricle	10.18 (10.07–10.28) (n = 9364)	10.53 (8.88–12.18) (n = 35)	B = -0.31 ± 1.16, β = −0.004, *P* = 0.791	0.99
Thalamus	7.22 (7.20–7.24) (n = 9422)	6.75 (6.45–7.04) (n = 36)	B = -0.28 ± 1.91, β = −0.022, *P* = 0.141	0.56
Caudate	3.31 (3.30–3.32) (n = 9406)	3.31 (3.20–3.42) (n = 36)	B = 0.0012 ± 0.091, β = 0.000, *P* = 0.989	0.99
Putamen	4.85 (4.84–4.86) (n = 9420)	4.96 (4.74–5.16) (n = 36)	B = 0.31 ± 0.14, β = 0.037, *P* = 0.033	0.53
Pallidum	1.24 (1.23–1.24) (n = 9419)	1.26 (1.20–1.33) (n = 36)	B = 0.092 ± 0.056, β = 0.030, *P* = 0.097	0.56
Hippocampus	4.08 (4.07–4.09) (n = 9409)	3.94 (3.76–4.12) (n = 36)	B = -0.056 ± 0.089, β = −0.010, *P* = 0.534	0.85
Amygdala	1.48 (1.48–1.49) (n = 9405)	1.47 (1.39–1.55) (n = 36)	B = 0.043 ± 0.048, β = 0.015, *P* = 0.367	0.73
Nucleus accumbens	4.96 (4.94–4.98) (n = 9419)	4.88 (4.57–5.20) (n = 36)	B = 0.023 ± 0.025, β = 0.016, *P* = 0.353	0.73
*Right Hemisphere*				
Lateral ventricle	9.45 (9.35–9.55) (n = 9351)	10.02 (8.60–11.44) (n = 35)	B = 0.20 ± 1.06, β = 0.003, *P* = 0.853	0.99
Thalamus	6.40 (6.39–6.41) (n = 9415)	6.21 (6.01–6.41) (n = 36)	B = -0.096 ± 0.119, β = −0.012, *P* = 0.420	0.75
Caudate	3.45 (3.44–3.46) (n = 9411)	3.43 (3.31–3.55) (n = 36)	B = 0.016 ± 0.094, β = 0.003, *P* = 0.862	0.99
Putamen	4.63 (4.62–4.65) (n = 9410)	4.63 (4.34–4.86) (n = 36)	B = 0.19 ± 0.12, β = 0.027, *P* = 0.118	0.56
Pallidum	1.43 (1.43–1.44) (n = 9395)	1.43 (1.39–1.48) (n = 36)	B = 0.019 ± 0.041, β = 0.008, *P* = 0.642	0.93
Hippocampus	4.22 (4.21–4.22) (n = 9410)	4.15 (4.00–4.32) (n = 36)	B = -0.097 ± 0.091, β = −0.017, *P* = 0.285	0.73
Amygdala	1.53 (1.52–1.53) (n = 9415)	1.50 (1.43–1.58) (n = 36)	B = 0.004 ± 0.047, β = 0.001, *P* = 0.938	0.99
Nucleus accumbens	5.22 (5.20–5.24) (n = 9421)	5.21 (4.88–5.54) (n = 36)	B = 0.021 ± 0.023, β = 0.016, *P* = 0.361	0.73

## Discussion

Here, we have systematically compared the prevalence/magnitude of a wide range of medical and neurobehavioural phenotypes in male and female carriers of small Xp22.31 duplications to that in sex-matched non-carrier controls, using a large adult sample drawn from the UK general population. As male and female duplication carriers did not differ from sex-matched controls across the vast majority of the large number of measures we assessed, our data indicate that within this sample (and presumably across a substantial proportion of the general population), such variants are generally benign. Previous data from clinical case studies/series have indicated that these microduplications are often associated with severe neurological and physical phenotypes. To resolve this apparent paradox we argue that in many previously-described cases the duplication may be an innocent bystander or a risk factor, and that other co-occurring genetic (e.g. alternative damaging copy number variants), environmental or stochastic factors acting in parallel with it predispose to the more severe phenotypes. Alternatively, it is possible that the duplication is pathogenic in a proportion of cases, and that these cases are not ascertained into the UK Biobank; individuals with developmental disorders/learning disabilities and other major health issues are under-represented in this sample (26).

The prevalence of Xp22.31 microduplications has been reported as 0.41% in general population controls (0.18% in males, and 0.52% in females) (18); the overall rate of 0.32% which we observed, in a sample > 80 times larger, is of comparable magnitude to this (equivalent to around 200 000 carriers in the UK, or > 23.5 million carriers worldwide). The 2–3 fold difference in prevalence of the variant between females and males in our sample, and in that of Liu *et al.* (18), most likely reflects the presence of two X chromosomes in females relative to one in males (hence double the opportunity for mutation), but could also partially reflect a less severe effect of duplication of this X-inactivation escaping-region in the former sex relative to the latter (i.e. a theoretical 50% increase in gene dosage in females compared to a 100% increase in gene dosage in males). The 3–4 fold higher prevalence of the duplication relative to the deletion (0.32% vs. < 0.1%) in the UK Biobank sample likely reflects the comparatively lower pathogenicity of the former variant.

We did identify a small number of phenotypes that differed significantly (*P* < 0.05) in prevalence and/or magnitude between groups. However, these comparisons did not survive stringent correction for multiple testing, were not statistically significant across both male and female duplication carrier groups, were associated with relatively small effect sizes, and were somewhat discrepant across objective and self-report measures. Hence, the positive results presented here will require replication in alternative large samples to test whether they represent true, or false positive, findings.

We have previously shown using the UK Biobank sample that deletion of the genomic region surrounding *STS* is associated with an increased rate of depressive-anxiety symptoms (but not diagnoses), and a reduction of the volume of sub-regions of the basal ganglia, including the left putamen (3). Our present data suggest that duplications of the same region, in both males and females, may impact upon similar phenotypes (though in the opposite direction), thus strengthening the evidence for a link between the associated genes and mood symptoms; the high expression of *STS* in the developing human basal ganglia (27), together with the enzyme’s regulatory role in neurochemical and steroid hormone-mediated mood disorder-related processes (28–30) implicates this gene/protein in particular. Elevated expression of *STS* in duplication carriers is expected to be associated with higher levels of circulating free steroids, including dehydroepiandrosterone (DHEA) and allopregnanolone; however, to our knowledge, free steroid levels in Xp22.31 duplication carriers have not yet been systematically evaluated. There is some evidence that administration of these free steroids may be beneficial in treating aspects of depression (29, 31, 32). Moreover, left putamen volume appears to be consistently smaller in older (33), first episode (34) and unmedicated (35) individuals with depression relative to unaffected controls. Hence, we propose that microduplications encompassing *STS* might protect against depressive-anxiety traits developmentally via increased free steroid abundance and/or increased putamen volume. The relationship between putamen volume and depression-related traits is likely to be complex, in that increased putamen volume has also been reported in individuals with psychotic disorder and associated mood symptoms (although this finding may be explained by the effects of antipsychotic administration) (36). Interestingly, males carrying duplications self-reported being diagnosed with mania/bipolar disorder more often than control males, although rates were low across both groups (<1%); there is some evidence that lower concentrations of circulating DHEAS (as might be expected in duplication carriers) are correlated with higher mania scores in a sample of patients with bipolar disorder (37). Hence, duplications could potentially have dissociable effects on depressive and manic traits, acting to reduce the former whilst eliciting the latter. In terms of neuroanatomy, duplication carriers also exhibited evidence for enlarged left lateral ventricle volume relative to controls; this finding aligns with a previous observation of a widened left lateral ventricle in a fetus possessing an Xp22.31 1.6 Mb microduplication (21).

Male duplication carriers were significantly more likely to have been diagnosed with inguinal hernia than male controls; the prevalence of this condition in our control group (6.8%) was similar to that previously observed in adult European males (7.2%) (38). Inguinal hernia has been reported in both Xp22.31 duplication (Supplementary Material, Table 1) and deletion (39, 40) cases, and approximately 20% of deletion cases are affected by the related condition of cryptorchidism (2, 41). Together, these findings suggest that aberrant (either over- or under-) expression of one of more genes in the interval of interest may predispose to aberrant inguinal canal anatomy and/or function. Abnormalities in the dosage of STS and consequent hormonal changes represent one plausible candidate mechanism underlying this association, although altered dosage of other genes within the interval may also be relevant (42); the respective contribution of these mechanisms remains to be investigated.

Female duplication carriers were more likely to have received a diagnosis of gastro-oesophageal reflux disease (without oesophagitis) than female controls. GORD and pyloric stenosis have previously been reported in male, but not female, duplication cases (18 and Supplementary Material, Table 1), and this may be consistent with our finding of increased proton pump inhibitor prescription in male duplication carriers. GORD is not a condition that is frequently associated with X-linked ichthyosis. Hence, the putative relationship between Xp22.31 gene dosage and GORD requires further investigation and clarification. Xp22.31 duplication was also associated with a higher self-reported rate of blistering/desquamating disorder diagnosis in females but not in males. This result may represent a true finding in that it is consistent with a role for genes within this interval (most likely *STS*) affecting skin morphology in a sex-biased manner, and with overexpression eliciting a similar phenotypic effect to under-expression.

In summary, our data indicate that Xp22.31 duplications are largely benign, with possible small protective effects against certain mood symptoms and small risk effects for specific medical conditions. Assuming replication of our positive findings, follow-up studies in carrier individuals and in model systems should clarify the underlying biological mechanisms. Documenting the full range of phenotypes associated with Xp22.31 duplications will be important to facilitate more informed counselling as genetic information for a greater proportion of the population becomes available.

## Materials and Methods

### Participants

Participants were individuals (40-69 yrs) recruited under UK Biobank informed consent procedures between 2006–2010, for which anonymised genotype/phenotype data were available.

### Copy Number Variant (CNV) calling

Anonymised genotype data were downloaded as raw (CEL) files from the UK Biobank website, and stored and processed as described previously (3). Individuals with duplications of 0.8–2.5 Mb spanning *STS* were identified, with calls and coordinates based upon the GRCh37/hg19 genome build. Following QC, CNV data were available for a total of 421 413 individuals. CNV data will be transferred to UK Biobank in accordance with their policies, and access to the data reported here may be obtained through application to UK Biobank.

### Measures

Hospital diagnoses according to the International Statistical Classification of Diseases and Related Health Problems Revision-10 (ICD-10, > 18 000 in total), self-reported non-cancer illnesses, relevant questions from the Mental Health Questionnaire (MHQ), and medication history were analysed. Highest levels of academic qualification and key performance measures on seven cognitive tasks (transformed and converted to z-scores) were analysed as described previously (3). Brain images were acquired using Siemens Skyra 3 T scanners in UK Biobank’s imaging centres in Cheadle and Newcastle, UK using identical acquisition protocols (43); T1-weighted brain images were processed using automated methods implemented in FreeSurfer (44) to obtain volumetric estimates for 8 right and left subcortical regions.

### Statistics

Data were analysed using SPSS v25.0 (IBM Corporation). As male and female duplication phenotypes could differ in magnitude and/or nature, two comparisons were performed: male duplication carriers vs. male non-carriers, and female duplication carriers vs. female non-carriers. Across the overall sample for each cognitive/neuroimaging measure, outlying values > 2.2 times the interquartile range below the first quartile, or above the third quartile, were excluded. Categorical data were analysed using Chi-squared Test (continuity-adjusted for 2x2 analyses)/Fisher Exact Test, with Odds Ratios and 95% confidence intervals presented as a measure of effect size. Ordinal/non-normally distributed data were compared using Mann–Whitney U test, with Cohen’s d presented as a measure of effect size. For cognitive and neuroanatomical analyses hierarchical linear regression with relevant covariates was performed. Data are presented as mean values with 95% confidence intervals or as mean values±standard error of the mean. Two-sided *P*-values<0.05 were regarded as nominally-significant, with *P*-values<0.1 after Benjamini-Hochberg adjustment regarded as surviving correction for multiple comparisons.

## Supplementary Material

Supplementary_Material_ddaa174

## Abbreviations

ADHD Attention Deficit Hyperactivity Disorder

CI Confidence interval

CNV Copy Number Variant(s)

DHEAS Dehydroepiandrosterone sulfate

GORD Gastro-oesophageal reflux disease

ICD-10 International Statistical Classification of Diseases and Related Health Problems Version 10

OR Odds Ratio

Mb Megabase

MHQ Mental Health Questionnaire

QC Quality Control

STS Steroid sulfatase

UK United Kingdom

XLI X-linked ichthyosis
