# Supplementary material for: Medical and neurobehavioural phenotypes in male and female carriers of Xp22.31 duplications in the UK Biobank
Source: Hum Mol Genet. 2020 Aug 7;29(17):2872–81. doi: 10.1093/hmg/ddaa174 (PMC7566349; doi:10.1093/hmg/ddaa174)
Supplement: Supplementary_Material_ddaa174 [file supplementary_material_ddaa174.docx]

**Supplementary Material**

**Supplementary Table 1.** Summary of phenotypes in open access patients with duplications spanning *STS* (0.8-2.5Mb, modal duplication size 1.56Mb) from DECIPHER database (accessed 14^th^ April 2020)(Firth, H.V., Richards, S.M., Bevan, A.P., Clayton, S., Corpas, M., Rajan, D., Van Vooren, S., Moreau, Y., Pettett, R.M., Carter, N.P. (2009) DECIPHER: Database of Chromosomal Imbalance and Phenotype in Humans using Ensembl Resources. *Am J Hum Genet,* **84**, 524-533.)

| **System affected** | **Phenotype** | **Male patients (n=50)** | **Female patients (n=52)** |
| --- | --- | --- | --- |
| **Brain and behaviour** | Aggressive behaviour | 1 | - |
|  | Autism/autistic behaviour | 8 | 5 |
|  | Emotional lability | 1 | - |
|  | ADHD/short attention span/hyperactivity | 2 | 3 |
|  | Delayed speech and language development | 7 | 3 |
|  | Intellectual disability | 21 | 20 |
|  | Chorea | 1 | - |
|  | Delayed gross motor development | 1 | - |
|  | Global developmental delay | 11 | 10 |
|  | Morphological abnormality of the central nervous system | 1 | - |
|  | Psychosis | 1 | - |
|  | Behavioural abnormality | 5 | - |
|  | Seizures/epileptic spasms | 5 | 5 |
|  | Sleep disturbance | 1 | - |
|  | Tremor | 1 | - |
|  | Ventriculomegaly | 1 | - |
|  | Cerebral palsy | 1 | - |
|  | Cognitive impairment | 1 | 3 |
|  | Cerebellar malformation | 1 | - |
|  | Holoprosencephaly | 1 | - |
|  | Abnormality of coordination/clumsiness | 2 | - |
|  | Dyslexia | 1 | - |
|  | Arachnoid cyst | - | 1 |
|  | Neurodevelopmental delay | - | 1 |
|  | Language impairment | - | 1 |
| **Head and face** | Cleft palate | 1 | 2 |
|  | Micrognathia | 1 | 1 |
|  | Trigonocephaly | 1 | - |
|  | Macrocephaly | 3 | - |
|  | Microcephaly | 4 | 4 |
|  | Abnormal facial shape | 2 | 2 |
|  | Low anterior hairline | 1 | - |
|  | Hypomimic face | 1 | - |
|  | Large forehead | 1 | - |
|  | Long philtrum | 1 | 1 |
|  | Telecanthus | 1 | - |
|  | Abnormality of the face | - | 2 |
|  | High palate | - | 1 |
|  | Coarse facial features | - | 1 |
|  | Malar flattening | - | 1 |
|  | Facial asymmetry | - | 1 |
|  | Hypertelorism | - | 1 |
| **Eyes, ears, nose and mouth** | Posteriorly-rotated ears | 2 | - |
|  | Anteverted nares | 1 | - |
|  | Strabismus | 3 | - |
|  | Large or cleft earlobe | 2 | - |
|  | Unilateral ptosis | 1 | - |
|  | Esotropia | 1 | - |
|  | Cupped ear | 1 | - |
|  | Deeply-set eye | 1 | - |
|  | Epicanthus | 1 | 2 |
|  | Hypermetropia | 1 | - |
|  | Narrow mouth | 1 | - |
|  | Congenital cataract | 1 | - |
|  | Long nose | 1 | - |
|  | Short upper lip | 1 | - |
|  | Low-set ears | 1 | - |
|  | Prominent or wide nasal bridge | 1 | 1 |
|  | Short nose | 1 | - |
|  | Thin ear helix | 1 | - |
|  | Macrodontia | - | 1 |
|  | Visual impairment/myopia | - | 4 |
|  | Nystagmus | - | 1 |
|  | Conductive hearing impairment | - | 1 |
|  | Protruding ear | - | 1 |
|  | Macroglossia | - | 1 |
|  | Up or down-slanted palpebral fissures | - | 2 |
|  | Widely-spaced teeth | - | 1 |
|  | Prominent ear helix | - | 1 |
|  | Sensorineural hearing impairment | - | 1 |
| **Reproductive** | Cryptorchidism | 2 | - |
|  | Decreased testicular size | 1 | - |
|  | Hypospadias | 2 | - |
|  | Early onset of sexual maturation | - | 1 |
|  | Premature birth | - | 1 |
| **Skeletal and growth** | Short stature | 2 | 1 |
|  | Delayed skeletal maturation | 1 | - |
|  | Delayed ossification of carpal bones | 1 | - |
|  | Tall stature | 1 | - |
|  | Joint hypermobility/laxity | 2 | 1 |
|  | Thoracolumbar scoliosis | 1 | - |
|  | Aplasia/hypoplasia involving bones of the lower limbs | - | 1 |
|  | Intrauterine growth retardation | - | 2 |
|  | Short long bone | - | 1 |
| **Hands and feet** | Deep palmar crease | 1 | - |
|  | Deep palmar crease | 1 | - |
|  | Small nail | 1 | - |
|  | Broad foot | 1 | - |
|  | Broad palm | 1 | - |
|  | Abnormality of thumb phalanx | 1 | - |
|  | Clinodactyly or shortness of the 5^th^ finger | 3 | 2 |
|  | Tapered finger | 1 | 1 |
|  | Joint contracture of the hand | 1 | - |
|  | Long fingers | 1 | - |
|  | Pes cavus | 1 | 1 |
|  | Pes planus | 1 | - |
|  | Short foot | - | 1 |
| **Digestive system** | Pyloric stenosis | 1 | - |
|  | Excessive salivation | 1 | - |
|  | Constipation | 2 | - |
|  | Anal atresia | 1 | - |
|  | Gastroesophageal reflux | 1 | - |
| **Skin** | Eczema/dry skin | 2 | - |
|  | Spotty hyperpigmentation | 2 | - |
|  | Abnormality of the skin | 1 | - |
|  | Café au lait spot | - | 1 |
|  | Atopic dermatitis | - | 1 |
| **Muscle** | Generalised hypotonia | 1 | - |
|  | Muscular hypotonia | 3 | 4 |
|  | Muscle fiber atrophy | 2 | - |
|  | Abnormality of the musculature | 1 | - |
|  | Neonatal hypotonia/failure to thrive or feeding difficulties in infancy | 2 | 1 |
|  | Diastasis recti | 1 | - |
| **Heart** | Ventricular septal defect | 1 | 2 |
|  | Cardiomegaly | 1 | - |
|  | Atrial septal defect | - | 3 |
|  | Congestive heart failure | - | 1 |
|  | Abnormal heart morphology | - | 2 |
| **Thyroid** | Hypothyroidism | 1 | - |
| **Immune** | Recurrent infections | 1 | - |
| **Kidney** | Enlarged kidney | - | 1 |
| **Other** | Sacral dimple | 1 | - |
|  | Congenital diaphragmatic hernia | 1 | - |
|  | Obesity | 1 | 2 |
|  | Frontal hirsutism | 1 | - |
|  | Supernumerary nipple | 1 | - |
|  | High-pitched voice/nasal speech | 1 | 1 |
|  | Inguinal hernia | 1 | - |
|  | Agenesis of pulmonary vessels | - | 1 |
|  | Increased nuchal translucency | - | 1 |
|  | Diabetes mellitus | - | 1 |
|  | Hypercalcemia | - | 1 |

**Supplementary Table 2.** ICD-10 related descriptive codes in male duplication carriers and male controls, occurring in >2.5% duplication carriers and >1.5% males overall.

| **UK Biobank diagnosis code** | **ICD-10 diagnosis** | **Male controls affected** | **Male controls unaffected** | **Male duplication carriers affected** | **Male duplication carriers unaffected** | **Prevalence in male controls** | **Prevalence in male duplication carriers** | **Statistical analysis** | **Benjamini-Hochberg corrected p-value (FDR<0.1)** |
| --- | --- | --- | --- | --- | --- | --- | --- | --- | --- |
| 4104 | I25.1 Atherosclerotic heart disease | 11479 | 181347 | 32 | 382 | 6.0 | 7.7 | Chi2[1]=2.021, p=0.155 | 0.407 |
| 4429 | I83.9 Varicose veins of lower extremities without ulcer or inflammation | 3240 | 189586 | 12 | 402 | 1.7 | 2.9 | Chi2[1]=3.006, p=0.083 | 0.407 |
| 4970 | K30 Dyspepsia | 3327 | 189499 | 11 | 403 | 1.7 | 2.7 | Chi2[1]=1.599, p=0.206 | 0.407 |
| 5004 | K40.9 Unilateral or unspecified inguinal hernia, without obstruction or gangrene | 13174 | 179652 | 39 | 375 | 6.8 | 9.4 | Chi2[1]=3.948, p=0.047 | 0.407 |
| 5029 | K44.9 Diaphragmatic hernia without obstruction or gangrene | 3722 | 189104 | 11 | 403 | 1.9 | 2.7 | Chi2[1]=0.800, p=0.371 | 0.536 |
| 5078 | K57.3 Diverticular disease of large intestine without perforation or abscess | 5707 | 187119 | 17 | 397 | 3.0 | 4.1 | Chi2[1]=1.512, p=0.219 | 0.407 |
| 5113 | K62.5 Haemorrhage of anus and rectum | 4466 | 188360 | 14 | 400 | 2.3 | 3.4 | Chi2[1]=1.627, p=0.202 | 0.407 |
| 5124 | K63.5 Polyp of colon | 4724 | 188502 | 14 | 400 | 2.4 | 3.4 | Chi2[1]=1.135, p=0.287 | 0.466 |
| 10399 | N40 Hyperplasia of prostate | 6041 | 186785 | 13 | 401 | 3.1 | 3.1 | Chi2[1]<0.000, p=>0.999 | >0.999 |
| 12306 | R07.4 Chest pain, unspecified | 10632 | 182194 | 26 | 388 | 5.5 | 6.3 | Chi2[1]=0.330, p=0.566 | 0.672 |
| 12388 | R31 Unspecified haematuria | 7567 | 185259 | 19 | 395 | 3.9 | 4.6 | Chi2[1]=0.324, p=0.569 | 0.672 |
| 12518 | R69 Unknown and unspecified causes of morbidity | 4283 | 188543 | 11 | 403 | 2.2 | 2.7 | Chi2[1]=0.188, p=0.664 | 0.719 |
| 18735 | Z46.6 Fitting and adjustment of urinary device | 3090 | 189736 | 11 | 403 | 1.6 | 2.7 | Chi2[1]=2.280, p=0.131 | 0.407 |

**Supplementary Table 3.** ICD-10 related descriptive codes in female duplication carriers and female controls, occurring in >2.5% duplication carriers and >1.5% females overall.

| **UK Biobank diagnosis code** | **ICD-10 diagnosis** | **Female controls affected** | **Female controls unaffected** | **Female duplication carriers affected** | **Female duplication carriers unaffected** | **Prevalence in female controls** | **Prevalence in female duplication carriers** | **Statistical analysis** | **Benjamini-Hochberg corrected p-value (FDR<0.1)** |
| --- | --- | --- | --- | --- | --- | --- | --- | --- | --- |
| 1484 | C50.9 Breast, unspecified | 7275 | 219960 | 31 | 907 | 3.2 | 3.3 | Chi2[1]=0.007, p=0.931 | 0.999 |
| 3401 | G56.0 Carpal tunnel syndrome | 6636 | 220599 | 35 | 903 | 2.9 | 3.7 | Chi2[1]=1.889, p=0.169 | 0.573 |
| 3704 | H26.9 Cataract, unspecified | 7124 | 220111 | 30 | 908 | 3.1 | 3.2 | Chi2[1]<0.000, p=0.986 | 0.999 |
| 4429 | I83.9 Varicose veins of lower extremities without ulcer or inflammation | 6925 | 220310 | 31 | 907 | 3.0 | 3.3 | Chi2[1]=0.131, p=0.717 | 0.999 |
| 4903 | K21.9 Gastro-oesophageal reflux disease without oesophagitis | 3630 | 223605 | 25 | 913 | 1.6 | 2.7 | Chi2[1]=4.884, p=0.027 | 0.432 |
| 4970 | K30 Dyspepsia | 6106 | 221129 | 28 | 910 | 2.7 | 3.0 | Chi2[1]=0.213, p=0.644 | 0.999 |
| 5078 | K57.3 Diverticular disease of large intestine without perforation or abscess | 7427 | 219808 | 28 | 910 | 3.3 | 3.0 | Chi2[1]=0.156, p=0.693 | 0.999 |
| 6562 | M16.9 Coxarthrosis, unspecified | 4297 | 222938 | 25 | 913 | 1.9 | 2.7 | Chi2[1]=2.611, p=0.106 | 0.573 |
| 6570 | M17.9 Gonarthrosis, unspecified | 5363 | 221872 | 24 | 914 | 2.4 | 2.6 | Chi2[1]=0.085, p=0.770 | 0.999 |
| 6637 | M20.1 Hallux valgus (acquired) | 5809 | 221426 | 31 | 907 | 2.6 | 3.3 | Chi2[1]=1.809, p=0.179 | 0.573 |
| 10557 | N84.0 Polyp of corpus uteri | 5956 | 221279 | 26 | 912 | 2.6 | 2.8 | Chi2[1]=0.035, p=0.852 | 0.999 |
| 10640 | N95.0 Postmenopausal bleeding | 7081 | 220154 | 29 | 909 | 3.1 | 3.1 | Chi2[1]<0.000, p=>0.999 | >0.999 |
| 12305 | R07.3 Other chest pain | 4326 | 222909 | 24 | 914 | 1.9 | 2.6 | Chi2[1]=1.806, p=0.179 | 0.573 |
| 12306 | R07.4 Chest pain, unspecified | 10070 | 217165 | 43 | 895 | 4.4 | 4.6 | Chi2[1]=0.022, p=0.883 | 0.999 |
| 12318 | R10.4 Other and unspecified abdominal pain | 8514 | 218721 | 36 | 902 | 3.7 | 3.8 | Chi2[1]=).004, p= 0.952 | 0.999 |
| 12518 | R69 Unknown and unspecified causes of morbidity | 5489 | 221746 | 29 | 909 | 2.4 | 3.1 | Chi2[1]=1.534, p=0.215 | 0.573 |

**Supplementary Table 4.** Mental health-related ICD-10 descriptive codes in male duplication carriers and male controls.

| **Class of disorder** | **Subclass of disorder** | **UK Biobank diagnosis code** | **ICD-10 diagnosis** | **Male controls affected** | **Male controls unaffected** | **Male duplication carriers affected** | **Male duplication carriers unaffected** | **Prevalence in male controls** | **Prevalence in male duplication carriers** | **P-value** |
| --- | --- | --- | --- | --- | --- | --- | --- | --- | --- | --- |
| **Developmental disorders** | Autism-related disorders | 3210 | Childhood autism |  |  |  |  |  |  |  |
|  |  | 3121 | Atypical autism |  |  |  |  |  |  |  |
|  |  | 3123 | Other childhood disintegrative disorder | 20 | 192806 | 0 | 414 | 0.0 | 0.0 | >0.99 |
|  |  | 3125 | Asperger's syndrome |  |  |  |  |  |  |  |
|  |  | 3126 | Other pervasive developmental disorders |  |  |  |  |  |  |  |
|  |  | 3127 | Pervasive developmental disorder, unspecified |  |  |  |  |  |  |  |
|  | Hyperkinetic disorder | 3131 | Disturbance of activity and attention |  |  |  |  |  |  |  |
|  |  | 3132 | Hyperkinetic conduct disorder | 0 | 192826 | 0 | 414 | 0.0 | 0.0 | >0.99 |
|  |  | 3133 | Other hyperkinetic disorders |  |  |  |  |  |  |  |
|  |  | 3134 | Hyperkinetic disorder, unspecified |  |  |  |  |  |  |  |
|  | Conduct disorders | 3136 | Conduct disorder confined to the family context |  |  |  |  |  |  |  |
|  |  | 3137 | Unsocialised conduct disorder |  |  |  |  |  |  |  |
|  |  | 3138 | Socialised conduct disorder |  |  |  |  |  |  |  |
|  |  | 3139 | Oppositional defiant disorder |  |  |  |  |  |  |  |
|  |  | 3140 | Other conduct disorders | 2 | 192824 | 0 | 414 | 0.0 | 0.0 | >0.99 |
|  |  | 3141 | Conduct disorder, unspecified |  |  |  |  |  |  |  |
|  |  | 3143 | Depressive conduct disorder |  |  |  |  |  |  |  |
|  |  | 3144 | Other mixed disorders of conduct and emotions |  |  |  |  |  |  |  |
|  |  | 3145 | Mixed disorder of conduct and emotions, unspecified |  |  |  |  |  |  |  |
|  | Dyslexia and alexia | 12446 | Dyslexia and alexia | 0 | 192826 | 0 | 414 | 0.0 | 0.0 | >0.99 |
| **Mood and anxiety disorders** | Mania/bipolar disorder | 2895 | Hypomania |  |  |  |  |  |  |  |
|  |  | 2896 | Mania without psychotic symptoms |  |  |  |  |  |  |  |
|  |  | 2897 | Mania with psychotic symptoms |  |  |  |  |  |  |  |
|  |  | 2898 | Other manic episodes |  |  |  |  |  |  |  |
|  |  | 2899 | Manic episode, unspecified |  |  |  |  |  |  |  |
|  |  | 2901 | Bipolar affective disorder, current episode hypomanic |  |  |  |  |  |  |  |
|  |  | 2902 | Bipolar affective disorder, current episode manic without psychotic symptoms |  |  |  |  |  |  |  |
|  |  | 2903 | Bipolar affective disorder, current episode manic with psychotic symptoms | 214 | 192612 | 2 | 412 | 0.1 | 0.5 | 0.127 |
|  |  | 2904 | Bipolar affective disorder, current episode mild or moderate depression |  |  |  |  |  |  |  |
|  |  | 2905 | Bipolar affective disorder, current episode severe depression without psychotic symptoms |  |  |  |  |  |  |  |
|  |  | 2906 | Bipolar affective disorder, current episode severe depression with psychotic symptoms |  |  |  |  |  |  |  |
|  |  | 2907 | Bipolar affective disorder, current episode mixed |  |  |  |  |  |  |  |
|  |  | 2908 | Bipolar affective disorder, currently in remission |  |  |  |  |  |  |  |
|  |  | 2909 | Other bipolar affective disorders |  |  |  |  |  |  |  |
|  |  | 2910 | Bipolar affective disorder, unspecified |  |  |  |  |  |  |  |
|  | Depressive disorder | 2912 | Mild depressive episode |  |  |  |  |  |  |  |
|  |  | 2913 | Moderate depressive episode |  |  |  |  |  |  |  |
|  |  | 2914 | Severe depressive episode without psychotic symptoms |  |  |  |  |  |  |  |
|  |  | 2915 | Severe depressive episode with psychotic symptoms |  |  |  |  |  |  |  |
|  |  | 2916 | Other depressive episodes |  |  |  |  |  |  |  |
|  |  | 2917 | Depressive episode, unspecified |  |  |  |  |  |  |  |
|  |  | 2919 | Recurrent depressive disorder, current episode mild | 591 | 192235 | 3 | 411 | 0.3 | 0.7 | 0.136 |
|  |  | 2920 | Recurrent depressive disorder, current episode moderate |  |  |  |  |  |  |  |
|  |  | 2921 | Recurrent depressive disorder, current episode severe without psychotic symptoms |  |  |  |  |  |  |  |
|  |  | 2922 | Recurrent depressive disorder, current episode severe with psychotic symptoms |  |  |  |  |  |  |  |
|  |  | 2923 | Recurrent depressive disorder, currently in remission |  |  |  |  |  |  |  |
|  |  | 2924 | Other recurrent depressive disorders |  |  |  |  |  |  |  |
|  |  | 2925 | Recurrent depressive disorder, unspecified |  |  |  |  |  |  |  |
|  | Anxiety disorder | 2937 | Agoraphobia |  |  |  |  |  |  |  |
|  |  | 2938 | Social phobias |  |  |  |  |  |  |  |
|  |  | 2939 | Specific (isolated) phobias |  |  |  |  |  |  |  |
|  |  | 2940 | Other phobic anxiety disorders |  |  |  |  |  |  |  |
|  |  | 2941 | Phobic anxiety disorder, unspecified |  |  |  |  |  |  |  |
|  |  | 2943 | Panic disorder [episodic paroxysmal anxiety] | 243 | 192583 | 0 | 414 | 0.1 | 0.0 | >0.99 |
|  |  | 2944 | Generalised anxiety disorder |  |  |  |  |  |  |  |
|  |  | 2945 | Mixed anxiety and depressive disorder |  |  |  |  |  |  |  |
|  |  | 2946 | Other mixed anxiety disorders |  |  |  |  |  |  |  |
|  |  | 2947 | Other specified anxiety disorders |  |  |  |  |  |  |  |
|  |  | 2948 | Anxiety disorder, unspecified |  |  |  |  |  |  |  |
|  | Obsessive Compulsive Disorder | 2950 | Predominantly obsessional thoughts or ruminations |  |  |  |  |  |  |  |
|  |  | 2951 | Predominantly compulsive acts [obsessional rituals] |  |  |  |  |  |  |  |
|  |  | 2952 | Mixed obsessional thoughts and acts | 19 | 192807 | 0 | 414 | 0.0 | 0.0 | >0.99 |
|  |  | 2953 | Other obsessive-compulsive disorders |  |  |  |  |  |  |  |
|  |  | 2954 | Obsessive-compulsive disorder, unspecified |  |  |  |  |  |  |  |

**Supplementary Table 5.** Mental health-related ICD-10 descriptive codes in female duplication carriers and female controls.

| **Class of disorder** | **Subclass of disorder** | **UK Biobank diagnosis code** | **ICD-10 diagnosis** | **Female controls affected** | **Female controls unaffected** | **Female duplication carriers affected** | **Female duplication carriers unaffected** | **Prevalence in female controls** | **Prevalence in female duplication carriers** | **P-value** |
| --- | --- | --- | --- | --- | --- | --- | --- | --- | --- | --- |
| **Developmental disorders** | Autism-related disorders | 3210 | Childhood autism |  |  |  |  |  |  |  |
|  |  | 3121 | Atypical autism |  |  |  |  |  |  |  |
|  |  | 3123 | Other childhood disintegrative disorder | 20 | 227215 | 0 | 938 | 0.0 | 0.0 | >0.99 |
|  |  | 3125 | Asperger's syndrome |  |  |  |  |  |  |  |
|  |  | 3126 | Other pervasive developmental disorders |  |  |  |  |  |  |  |
|  |  | 3127 | Pervasive developmental disorder, unspecified |  |  |  |  |  |  |  |
|  | Hyperkinetic disorder | 3131 | Disturbance of activity and attention |  |  |  |  |  |  |  |
|  |  | 3132 | Hyperkinetic conduct disorder | 0 | 227235 | 0 | 938 | 0.0 | 0.0 | >0.99 |
|  |  | 3133 | Other hyperkinetic disorders |  |  |  |  |  |  |  |
|  |  | 3134 | Hyperkinetic disorder, unspecified |  |  |  |  |  |  |  |
|  | Conduct disorders | 3136 | Conduct disorder confined to the family context |  |  |  |  |  |  |  |
|  |  | 3137 | Unsocialised conduct disorder |  |  |  |  |  |  |  |
|  |  | 3138 | Socialised conduct disorder |  |  |  |  |  |  |  |
|  |  | 3139 | Oppositional defiant disorder |  |  |  |  |  |  |  |
|  |  | 3140 | Other conduct disorders | 0 | 227235 | 0 | 938 | 0.0 | 0.0 | >0.99 |
|  |  | 3141 | Conduct disorder, unspecified |  |  |  |  |  |  |  |
|  |  | 3143 | Depressive conduct disorder |  |  |  |  |  |  |  |
|  |  | 3144 | Other mixed disorders of conduct and emotions |  |  |  |  |  |  |  |
|  |  | 3145 | Mixed disorder of conduct and emotions, unspecified |  |  |  |  |  |  |  |
|  | Dyslexia and alexia | 12446 | Dyslexia and alexia | 2 | 227233 | 0 | 938 | 0.0 | 0.0 | >0.99 |
| **Mood and anxiety disorders** | Mania/bipolar disorder | 2895 | Hypomania |  |  |  |  |  |  |  |
|  |  | 2896 | Mania without psychotic symptoms |  |  |  |  |  |  |  |
|  |  | 2897 | Mania with psychotic symptoms |  |  |  |  |  |  |  |
|  |  | 2898 | Other manic episodes |  |  |  |  |  |  |  |
|  |  | 2899 | Manic episode, unspecified |  |  |  |  |  |  |  |
|  |  | 2901 | Bipolar affective disorder, current episode hypomanic |  |  |  |  |  |  |  |
|  |  | 2902 | Bipolar affective disorder, current episode manic without psychotic symptoms |  |  |  |  |  |  |  |
|  |  | 2903 | Bipolar affective disorder, current episode manic with psychotic symptoms | 344 | 226891 | 2 | 936 | 0.2 | 0.2 | 0.656 |
|  |  | 2904 | Bipolar affective disorder, current episode mild or moderate depression |  |  |  |  |  |  |  |
|  |  | 2905 | Bipolar affective disorder, current episode severe depression without psychotic symptoms |  |  |  |  |  |  |  |
|  |  | 2906 | Bipolar affective disorder, current episode severe depression with psychotic symptoms |  |  |  |  |  |  |  |
|  |  | 2907 | Bipolar affective disorder, current episode mixed |  |  |  |  |  |  |  |
|  |  | 2908 | Bipolar affective disorder, currently in remission |  |  |  |  |  |  |  |
|  |  | 2909 | Other bipolar affective disorders |  |  |  |  |  |  |  |
|  |  | 2910 | Bipolar affective disorder, unspecified |  |  |  |  |  |  |  |
|  | Depressive disorder | 2912 | Mild depressive episode |  |  |  |  |  |  |  |
|  |  | 2913 | Moderate depressive episode |  |  |  |  |  |  |  |
|  |  | 2914 | Severe depressive episode without psychotic symptoms |  |  |  |  |  |  |  |
|  |  | 2915 | Severe depressive episode with psychotic symptoms |  |  |  |  |  |  |  |
|  |  | 2916 | Other depressive episodes |  |  |  |  |  |  |  |
|  |  | 2917 | Depressive episode, unspecified |  |  |  |  |  |  |  |
|  |  | 2919 | Recurrent depressive disorder, current episode mild | 758 | 226477 | 2 | 936 | 0.3 | 0.2 | 0.776 |
|  |  | 2920 | Recurrent depressive disorder, current episode moderate |  |  |  |  |  |  |  |
|  |  | 2921 | Recurrent depressive disorder, current episode severe without psychotic symptoms |  |  |  |  |  |  |  |
|  |  | 2922 | Recurrent depressive disorder, current episode severe with psychotic symptoms |  |  |  |  |  |  |  |
|  |  | 2923 | Recurrent depressive disorder, currently in remission |  |  |  |  |  |  |  |
|  |  | 2924 | Other recurrent depressive disorders |  |  |  |  |  |  |  |
|  |  | 2925 | Recurrent depressive disorder, unspecified |  |  |  |  |  |  |  |
|  | Anxiety disorder | 2937 | Agoraphobia |  |  |  |  |  |  |  |
|  |  | 2938 | Social phobias |  |  |  |  |  |  |  |
|  |  | 2939 | Specific (isolated) phobias |  |  |  |  |  |  |  |
|  |  | 2940 | Other phobic anxiety disorders |  |  |  |  |  |  |  |
|  |  | 2941 | Phobic anxiety disorder, unspecified |  |  |  |  |  |  |  |
|  |  | 2943 | Panic disorder [episodic paroxysmal anxiety] | 366 | 226869 | 0 | 938 | 0.2 | 0.0 | 0.413 |
|  |  | 2944 | Generalised anxiety disorder |  |  |  |  |  |  |  |
|  |  | 2945 | Mixed anxiety and depressive disorder |  |  |  |  |  |  |  |
|  |  | 2946 | Other mixed anxiety disorders |  |  |  |  |  |  |  |
|  |  | 2947 | Other specified anxiety disorders |  |  |  |  |  |  |  |
|  |  | 2948 | Anxiety disorder, unspecified |  |  |  |  |  |  |  |
|  | Obsessive Compulsive Disorder | 2950 | Predominantly obsessional thoughts or ruminations |  |  |  |  |  |  |  |
|  |  | 2951 | Predominantly compulsive acts [obsessional rituals] |  |  |  |  |  |  |  |
|  |  | 2952 | Mixed obsessional thoughts and acts | 14 | 227221 | 0 | 938 | 0.0 | 0.0 | >0.99 |
|  |  | 2953 | Other obsessive-compulsive disorders |  |  |  |  |  |  |  |
|  |  | 2954 | Obsessive-compulsive disorder, unspecified |  |  |  |  |  |  |  |

**Supplementary Table 6.** Self-reported non-cancer diagnoses in male duplication carriers and male controls.

| **Body system** | **UK Biobank non-cancer illness code** | **Non-cancer illness** | **Male controls affected** | **Male controls unaffected** | **Male duplication carriers affected** | **Male duplication carriers unaffected** | **Prevalence in male controls** | **Prevalence in male duplication carriers** | **P-value** |
| --- | --- | --- | --- | --- | --- | --- | --- | --- | --- |
|  |  |  |  |  |  |  |  |  |  |
| Heart and cardiovascular | 1066 | Heart/cardiac problem | 696 | 192130 | 3 | 411 | 0.4 | 0.7 | 0.190 |
|  | 1077 | Heart arrhythmia | 1180 | 191646 | 4 | 410 | 0.6 | 1.0 | 0.328 |
|  | 1471 | Atrial fibrillation | 2385 | 190441 | 2 | 412 | 1.2 | 0.5 | Chi2[1]=1.356, p=0.244 |
|  | 1483 | Atrial flutter | 73 | 192753 | 0 | 414 | 0.0 | 0.0 | >0.999 |
|  | 1485 | Irregular heartbeat | 415 | 192411 | 1 | 413 | 0.2 | 0.2 | 0.591 |
|  |  |  |  |  |  |  |  |  |  |
| Reproductive | 1214 | Testicular problems (not cancer) | 599 | 192227 | 1 | 413 | 0.3 | 0.2 | >0.999 |
|  | 1404 | Male infertility | 27 | 192799 | 0 | 414 | 0.0 | 0.0 | >0.999 |
|  | 1679 | Undescended testicle | 192 | 192634 | 0 | 414 | 0.1 | 0.0 | >0.999 |
|  |  |  |  |  |  |  |  |  |  |
| Thyroid | 1224 | Thyroid problem (not cancer) | 163 | 192663 | 1 | 413 | 0.1 | 0.2 | 0.297 |
|  | 1225 | Hyperthyroidism | 599 | 192227 | 0 | 414 | 0.3 | 0.0 | 0.644 |
|  | 1226 | Hypothyroidism | 3141 | 189685 | 5 | 409 | 1.6 | 1.2 | Chi2[1]=0.232, p=0.630 |
|  |  |  |  |  |  |  |  |  |  |
| Eye | 1278 | Cataract | 3022 | 189804 | 7 | 407 | 1.6 | 1.7 | Chi2[1]=0.000, p=0.997 |
|  |  |  |  |  |  |  |  |  |  |
| Immune system | 1374 | Allergy/hypersensitivity/anaphylaxis | 1028 | 191798 | 2 | 412 | 0.5 | 0.5 | >0.999 |
|  |  |  |  |  |  |  |  |  |  |
| Hair | 1667 | Alopecia/hair loss | 46 | 192780 | 0 | 414 | 0.0 | 0.0 | >0.999 |
|  |  |  |  |  |  |  |  |  |  |
| Skin | 1452 | Eczema/dermatitis | 5183 | 187643 | 7 | 407 | 2.7 | 1.7 | Chi2[1]=1.213, p=0.271 |
|  | 1453 | Psoriasis | 2660 | 190166 | 6 | 408 | 1.4 | 1.4 | Chi2[1]=0.000, p>0.999 |
|  | 1454 | Blistering/desquamating skin disorder | 330 | 192496 | 2 | 412 | 0.2 | 0.5 | 0.16 |
|  |  |  |  |  |  |  |  |  |  |
| Nervous system | 1243 | Psychological/psychiatric problem | 175 | 192651 | 0 | 414 | 0.1 | 0.0 | >0.999 |
|  | 1258 | Chronic/degenerative neurological problem | 67 | 192759 | 0 | 414 | 0.0 | 0.0 | >0.999 |
|  | 1259 | Motor neurone disease | 36 | 192790 | 0 | 414 | 0.0 | 0.0 | >0.999 |
|  | 1261 | Multiple sclerosis | 394 | 192432 | 0 | 414 | 0.2 | 0.0 | >0.999 |
|  | 1262 | Parkinson's Disease | 488 | 192338 | 1 | 413 | 0.3 | 0.2 | >0.999 |
|  | 1263 | Dementia/Alzheimer's/cognitive impairment | 65 | 192761 | 0 | 414 | 0.0 | 0.0 | >0.999 |
|  | 1264 | Epilepsy | 1734 | 191092 | 6 | 408 | 0.9 | 1.4 | 0.285 |
|  | 1265 | Migraine | 2933 | 189893 | 7 | 407 | 1.5 | 1.7 | Chi2[1]=0.007, p=0.936 |
|  | 1286 | Depression | 8668 | 184158 | 12 | 402 | 4.5 | 2.9 | Chi2[1]=2.097, p=0.148 |
|  | 1287 | Anxiety/panic attacks | 2149 | 190677 | 7 | 407 | 1.1 | 1.7 | 0.238 |
|  | 1288 | Nervous breakdown | 279 | 192547 | 1 | 413 | 0.1 | 0.2 | 0.452 |
|  | 1289 | Schizophrenia | 308 | 192518 | 1 | 413 | 0.2 | 0.2 | 0.485 |
|  | 1290 | Deliberate self-harm/suicide | 84 | 192742 | 0 | 414 | 0.0 | 0.0 | >0.999 |
|  | 1291 | Mania/bipolar disorder | 514 | 192312 | 4 | 410 | 0.3 | 1.0 | 0.026 |
|  | 1408 | Alcohol dependency | 413 | 192355 | 1 | 413 | 0.2 | 0.2 | >0.999 |
|  | 1409 | Opioid dependency | 21 | 192805 | 0 | 414 | 0.0 | 0.0 | >0.999 |
|  | 1410 | Other substance abuse | 34 | 192792 | 1 | 413 | 0.0 | 0.2 | 0.072 |
|  | 1469 | Posttraumatic Stress Disorder | 145 | 192681 | 1 | 413 | 0.1 | 0.2 | 0.269 |
|  | 1470 | Anorexia/bulimia | 16 | 192810 | 0 | 414 | 0.0 | 0.0 | >0.999 |
|  | 1531 | Postnatal depression | 1 | 192825 | 0 | 414 | 0.0 | 0.0 | >0.999 |
|  | 1614 | Stress | 272 | 192554 | 0 | 414 | 0.1 | 0.0 | >0.999 |
|  | 1615 | Obsessive Compulsive Disorder | 52 | 192774 | 0 | 414 | 0.0 | 0.0 | >0.999 |
|  | 1616 | Insomnia | 121 | 192705 | 0 | 414 | 0.1 | 0.0 | >0.999 |
|  |  |  |  |  |  |  |  |  |  |
| Gastrointestinal | 1134 | Oesophageal disorder | 117 | 192709 | 1 | 413 | 0.1 | 0.2 | 0.224 |
|  | 1135 | Stomach disorder | 473 | 192353 | 2 | 412 | 0.2 | 0.5 | 0.271 |
|  | 1137 | Other abdominal problem | 209 | 192617 | 0 | 414 | 0.1 | 0.0 | >0.999 |
|  | 1138 | Gastro-oesophageal reflux (GORD) / gastric reflux | 9007 | 183819 | 25 | 389 | 4.7 | 6.0 | Chi2[1]=1.441, p=0.196 |
|  | 1139 | Oesophagitis/Barrett’s oesophagus | 769 | 192057 | 1 | 413 | 0.4 | 0.2 | >0.999 |
|  | 1140 | Oesophageal stricture | 58 | 192768 | 0 | 414 | 0.0 | 0.0 | >0.999 |
|  | 1142 | Gastric/stomach ulcers | 1792 | 191034 | 3 | 411 | 0.9 | 0.7 | >0.999 |
|  | 1143 | Gastritis/gastric erosions | 311 | 192515 | 1 | 413 | 0.2 | 0.2 | 0.488 |
|  | 1400 | Peptic ulcer | 265 | 192561 | 0 | 414 | 0.1 | 0.0 | >0.999 |
| Hernia | 1474 | Hiatus hernia | 4161 | 188665 | 12 | 402 | 2.2 | 2.9 | Chi2[1]=0.751, p=0.386 |
|  | 1512 | Umbilical hernia | 255 | 192571 | 0 | 414 | 0.1 | 0.0 | >0.999 |
|  | 1513 | Inguinal hernia | 1820 | 191006 | 8 | 406 | 0.9 | 1.9 | 0.065 |
|  | 1605 | Femoral hernia | 52 | 192774 | 0 | 414 | 0.0 | 0.0 | >0.999 |
|  | 1606 | Incisional hernia | 40 | 192786 | 0 | 414 | 0.0 | 0.0 | >0.999 |

**Supplementary Table 7.** Self-reported non-cancer diagnoses in female duplication carriers and female controls.

| **Body system** | **UK Biobank non-cancer illness code** | **Non-cancer illness** | **Female controls affected** | **Female controls unaffected** | **Female duplication carriers affected** | **Female duplication carriers unaffected** | **Prevalence in female controls** | **Prevalence in female duplication carriers** | **P-value** |
| --- | --- | --- | --- | --- | --- | --- | --- | --- | --- |
|  |  |  |  |  |  |  |  |  |  |
| Heart and cardiovascular | 1066 | Heart/cardiac problem | 664 | 226571 | 4 | 934 | 0.3 | 0.4 | 0.359 |
|  | 1077 | Heart arrhythmia | 1142 | 226093 | 7 | 931 | 0.5 | 0.7 | 0.248 |
|  | 1471 | Atrial fibrillation | 1080 | 226155 | 4 | 934 | 0.5 | 0.4 | >0.999 |
|  | 1483 | Atrial flutter | 26 | 227209 | 0 | 938 | 0.0 | 0.0 | >0.999 |
|  | 1485 | Irregular heartbeat | 389 | 226846 | 0 | 938 | 0.2 | 0.0 | 0.417 |
|  |  |  |  |  |  |  |  |  |  |
| Reproductive | 1402 | Endometriosis | 3539 | 223696 | 18 | 920 | 1.6 | 1.9 | Chi2[1]=0.578, p=0.447 |
|  | 1403 | Female infertility | 512 | 226723 | 5 | 933 | 0.2 | 0.5 | 0.064 |
|  |  |  |  |  |  |  |  |  |  |
| Thyroid | 1224 | Thyroid problem (not cancer) | 943 | 226292 | 4 | 934 | 0.4 | 0.4 | 0.799 |
|  | 1225 | Hyperthyroidism | 2691 | 224544 | 9 | 929 | 1.2 | 1.0 | Chi2[1]=0.234, p=0.628 |
|  | 1226 | Hypothyroidism | 17852 | 209383 | 75 | 863 | 7.9 | 8.0 | Chi2[1]=0.010, p=0.922 |
|  |  |  |  |  |  |  |  |  |  |
| Eye | 1278 | Cataract | 3916 | 223319 | 11 | 927 | 1.7 | 1.2 | Chi2[1]=1.365, p=0.243 |
|  |  |  |  |  |  |  |  |  |  |
| Immune system | 1374 | Allergy/hypersensitivity/anaphylaxis | 2028 | 225207 | 8 | 930 | 0.9 | 0.9 | Chi2[1]=0.000, p>0.999 |
|  |  |  |  |  |  |  |  |  |  |
| Hair | 1667 | Alopecia/hair loss | 66 | 227169 | 0 | 938 | 0.0 | 0.0 | >0.999 |
|  |  |  |  |  |  |  |  |  |  |
| Skin | 1452 | Eczema/dermatitis | 6236 | 220999 | 30 | 908 | 2.7 | 3.2 | Chi2[1]=0.561, p=0.454 |
|  | 1453 | Psoriasis | 2319 | 224916 | 10 | 928 | 1.0 | 1.1 | Chi2[1]=0.000, p.0.999 |
|  | 1454 | Blistering/desquamating skin disorder | 331 | 226904 | 5 | 933 | 0.1 | 0.5 | 0.013 |
|  |  |  |  |  |  |  |  |  |  |
| Nervous system | 1243 | Psychological/psychiatric problem | 218 | 227017 | 1 | 937 | 0.1 | 0.1 | 0.594 |
|  | 1258 | Chronic/degenerative neurological problem | 80 | 227155 | 1 | 937 | 0.0 | 0.1 | 0.284 |
|  | 1259 | Motor neurone disease | 14 | 227221 | 0 | 938 | 0.0 | 0.0 | >0.999 |
|  | 1261 | Multiple sclerosis | 1133 | 226102 | 5 | 933 | 0.5 | 0.5 | 0.814 |
|  | 1262 | Parkinson's Disease | 282 | 226953 | 2 | 936 | 0.1 | 0.2 | 0.326 |
|  | 1263 | Dementia/Alzheimer's/cognitive impairment | 51 | 227184 | 0 | 938 | 0.0 | 0.0 | >0.999 |
|  | 1264 | Epilepsy | 1773 | 225462 | 5 | 933 | 0.8 | 0.5 | 0.573 |
|  | 1265 | Migraine | 10052 | 217183 | 38 | 900 | 4.4 | 4.1 | Chi2[1]=0.225, p=0.635 |
|  | 1286 | Depression | 16458 | 210777 | 65 | 873 | 7.2 | 6.9 | Chi2[1]=0.094, p=0.760 |
|  | 1287 | Anxiety/panic attacks | 3951 | 223284 | 14 | 924 | 1.7 | 1.5 | Chi2[1]=0.203, p=0.652 |
|  | 1288 | Nervous breakdown | 366 | 226869 | 0 | 938 | 0.2 | 0.0 | 0.413 |
|  | 1289 | Schizophrenia | 147 | 227088 | 0 | 938 | 0.1 | 0.0 | >0.999 |
|  | 1290 | Deliberate self-harm/suicide | 109 | 227126 | 1 | 937 | 0.0 | 0.1 | 0.364 |
|  | 1291 | Mania/bipolar disorder | 656 | 226579 | 2 | 936 | 0.3 | 0.2 | >0.999 |
|  | 1408 | Alcohol dependency | 177 | 227058 | 0 | 938 | 0.1 | 0.0 | >0.999 |
|  | 1409 | Opioid dependency | 5 | 227230 | 0 | 938 | 0.0 | 0.0 | >0.999 |
|  | 1410 | Other substance abuse | 14 | 227221 | 0 | 938 | 0.0 | 0.0 | >0.999 |
|  | 1469 | Posttraumatic Stress Disorder | 150 | 227085 | 0 | 938 | 0.1 | 0.0 | >0.999 |
|  | 1470 | Anorexia/bulimia | 313 | 226922 | 1 | 937 | 0.1 | 0.1 | >0.999 |
|  | 1531 | Postnatal depression | 399 | 226834 | 0 | 938 | 0.2 | 0.0 | 0.425 |
|  | 1614 | Stress | 375 | 226860 | 1 | 937 | 0.2 | 0.1 | >0.999 |
|  | 1615 | Obsessive Compulsive Disorder | 48 | 227187 | 0 | 938 | 0.0 | 0.0 | >0.999 |
|  | 1616 | Insomnia | 295 | 226940 | 1 | 937 | 0.1 | 0.1 | >0.999 |
|  |  |  |  |  |  |  |  |  |  |
| Gastrointestinal | 1134 | Oesophageal disorder | 141 | 227094 | 1 | 937 | 0.1 | 0.1 | 0.443 |
|  | 1135 | Stomach disorder | 447 | 226788 | 1 | 937 | 0.2 | 0.1 | >0.999 |
|  | 1137 | Other abdominal problem | 293 | 226942 | 0 | 938 | 0.1 | 0.0 | 0.638 |
|  | 1138 | Gastro-oesophageal reflux (GORD) / gastric reflux | 10013 | 217222 | 38 | 900 | 4.4 | 4.1 | Chi2[1]=0.202, p=0.633 |
|  | 1139 | Oesophagitis/Barrett’s oesophagus | 583 | 226652 | 2 | 936 | 0.3 | 0.2 | >0.999 |
|  | 1140 | Oesophageal stricture | 40 | 227195 | 0 | 938 | 0.0 | 0.0 | >0.999 |
|  | 1142 | Gastric/stomach ulcers | 1290 | 225945 | 7 | 931 | 0.6 | 0.7 | Chi2[1]=0.258, p=0.611 |
|  | 1143 | Gastritis/gastric erosions | 397 | 226838 | 4 | 934 | 0.2 | 0.4 | 0.085 |
|  | 1400 | Peptic ulcer | 171 | 227064 | 0 | 938 | 0.1 | 0.0 | >0.999 |
|  |  |  |  |  |  |  |  |  |  |
| Hernia | 1474 | Hiatus hernia | 5926 | 221309 | 26 | 912 | 2.6 | 2.8 | Chi2[1]=0.045, p=0.832 |
|  | 1512 | Umbilical hernia | 146 | 227089 | 1 | 937 | 0.1 | 0.1 | 0.454 |
|  | 1513 | Inguinal hernia | 174 | 227061 | 1 | 937 | 0.1 | 0.1 | 0.514 |
|  | 1605 | Femoral hernia | 67 | 227168 | 1 | 937 | 0.0 | 0.1 | 0.244 |
|  | 1606 | Incisional hernia | 48 | 227187 | 0 | 938 | 0.0 | 0.0 | >0.999 |

**Supplementary Table 8.** Mental Health Questionnaire (MHQ) responses in male duplication carriers and male controls.

| **Depression** |  |  |  |
| --- | --- | --- | --- |
| *Dual answer questions* | **Male control** | **Male duplication** | **Statistical analysis** |
| Ever had prolonged feelings of sadness or depression? (Yes/No) | 25955/33327 | 44/85 | Chi2[1]=4.509, p=0.034 |
| Ever had prolonged loss of interest in normal activities? (Yes/No) | 18679/40601 | 35/94 | Chi2[1]=0.949, p=0.330 |
| Depression possibly related to stressful or traumatic event (Yes/No) | 17565/9856 | 28/19 | Chi2[1]=0.238, p=0.626 |
| Feelings of tiredness during worst episode of depression (Yes/No) | 18607/5964 | 28/14 | Chi2[1]=1.412, p=0.235 |
| Did your sleep change? (Yes/No) | 17557/6136 | 26/14 | Chi2[1]=1.282, p=0.258 |
| Trouble falling asleep (Yes/No) | 13394/4163 | 17/9 | Chi2[1]=1.156, p=0.282 |
| Sleeping too much (Yes/No) | 3460/14097 | 7/19 | Chi2[1]=0.459, p=0.498 |
| Waking too early (Yes/No) | 13224/4333 | 17/9 | Chi2[1]=0.896, p=0.344 |
| Difficulty concentrating during worst depression (Yes/No) | 18675/6063 | 33/12 | Chi2[1]=0.026, p=0.871 |
| Feelings of worthlessness during worst episode of depression (Yes/No) | 12492/13356 | 14/30 | Chi2[1]=4.157, p=0.041 |
| Thoughts of death during worst depression (Yes/No) | 12041/14039 | 16/30 | Chi2[1]=1.959, p=0.162 |
| Depression possibly related to childbirth (Yes/No) | N/A | N/A | N/A |
| Professional informed about depression (Yes/No) | 15540/11876 | 29/19 | Chi2[1]=0.141, p=0.707 |
| Substances taken for depression (unprescribed)(Yes/No) | 1253/58165 | 2/127 | p>0.999 |
| Substances taken for depression (prescribed)(Yes/No) | 10021/49397 | 18/111 | Chi2[1]=0.585, p=0.444 |
| Substances taken for depression (drugs or alcohol)(Yes/No) | 4609/54809 | 7/122 | Chi2[1]=0.679, p=0.410 |
| Talking therapies (Yes/No) | 9150/50268 | 11/18 | Chi2[1]=4.157, p=0.041 |
| Other non-drug therapies (e.g. yoga) (Yes/No) | 2365/57053 | 3/126 | Chi2[1]=0.540, p=0.462 |
| *Multiple answer questions* |  |  |  |
| Trouble falling or staying asleep, or sleeping too much (1: not at all-4:nearly every day) | 1.60±0.004 | 1.59±0.078 | U=3717693, p=0.656 |
| Recent feelings of inadequacy (1: not at all-4:nearly every day) | 1.22±0.002 | 1.16±0.044 | U=3614172, p=0.174 |
| Recent trouble concentrating on things (1: not at all-4:nearly every day) | 1.23±0.002 | 1.22±0.052 | U=3781213, p=0.732 |
| Recent feelings of depression (1: not at all-4:nearly every day) | 1.24±0.002 | 1.17±0.043 | U=3581007, p=0.074 |
| Recent poor appetite or overeating (1: not at all-4:nearly every day) | 1.19±0.002 | 1.12±0.033 | U=3719796, p=0.340 |
| Recent thoughts of suicide or self-harm (1: not at all-4:nearly every day) | 1.06±0.001 | 1.02±0.11 | U=3698919, p=0.114 |
| Recent lack of interest or pleasure in doing things (1: not at all-4:nearly every day) | 1.24±0.002 | 1.15±0.040 | U=3571583, p=0.053 |
| Recent changes in speed/amount of moving or speaking (1: not at all-4:nearly every day) | 1.07±0.001 | 1.04±0.017 | U=3765120, p=0.379 |
| Recent feelings of tiredness or low energy (1: not at all-4:nearly every day) | 1.60±0.003 | 1.45±0.060 | U=3398374, p=0.022 |
| Fraction of day affected during worst episode of depression (1:less than half the day-4:all day long) | 2.73±0.006 | 2.71±0.144 | U=572321, p=0.870 |
| Frequency of depressed days during worst episode of depression (1:less often-3:every day) | 2.18±0.004 | 2.09±0.087 | U=563287, p=0.301 |
| Duration of worst depression (1:less than a month-6:over two years) | 2.86±0.009 | 2.62±0.242 | U=0.566419, p=0.168 |
| Impact on normal roles during worst episode of depression (0:not at all-3:a lot) | 1.89±0.006 | 1.94±0.141 | U=641301, p=0.730 |
| Age at first episode of depression (yrs) | 39.24±0.096 | 41.40±2.515 | U=499122, p=0.395 |
| Age at last episode of depression (yrs) | 49.64±0.085 | 47.91±2.338 | U=495459.5, p=0.529 |
| Lifetime number of depressed periods | 167.11±2.283 | 107.89±45.334 | U=525226.5, p=0.049 |
| **Mania** |  |  |  |
| *Dual answer questions* | **Male control** | **Male duplication** | **Statistics** |
| Manifestations of mania (more talkative than usual) (Yes/No) | 2945/56473 | 7/122 | Chi2[1]=0.002, p=0.966 |
| Manifestations of mania (more restless than usual)(Yes/No) | 6687/52731 | 15/114 | Chi2[1]=0.000, p>0.999 |
| Manifestations of mania (my thoughts were racing)(Yes/No) | 5411/54007 | 8/121 | Chi2[1]=0.986, p=0.321 |
| Manifestations of mania (needed less sleep than usual)(Yes/No) | 2008/57410 | 3/126 | p=0.804 |
| Manifestations of mania (more creative than usual)(Yes/No) | 1929/57489 | 4/125 | p>0.999 |
| Manifestations of mania (was easily distracted)(Yes/No) | 4602/54816 | 9/120 | Chi2[1]=0.026, p=0.872 |
| Manifestations of mania (was more confident than usual)(Yes/No) | 2013/57405 | 4/125 | P>0.999 |
| Manifestations of mania (was more active than usual)(Yes/No) | 3059/56359 | 7/122 | Chi2[1]=0.000, p>0.999 |
| Ever had a period of mania/excitability? (Yes/No) | 2849/54887 | 8/120 | Chi2[1]=0.232, p=0.630 |
| Ever had a period of extreme irritability? (Yes/No) | 14407/43389 | 22/105 | Chi2[1]=3.522, p=0.061 |
| Severity of problems due to mania or irritability (No problems/caused problems) | 4222/9085 | 11/12 | Chi2[1]=2.053, p=0.152 |
| *Multiple answer questions* |  |  |  |
| Longest period of mania or irritability (1: less than 24hrs-3: a week or more) | 1.86±0.007 | 1.84±0.160 | U=169453, p=0.863 |
| **Anxiety** |  |  |  |
| *Dual answer questions* |  |  |  |
| Ever felt worried, tense or anxious for most of a month or longer (Yes/No) | 11954/44648 | 19/108 | Chi2[1]=2.528, p=0.112 |
| Ever worried more than most people would in similar situation (Yes/No) | 10887/39743 | 19/96 | Chi2[1]=1.405, p=0.236 |
| Stronger worrying (than other people) during period of worst anxiety (Yes/No) | 8690/1600 | 14/1 | Chi2[1]=0.351, p=0.554 |
| Worried most days during period of worst anxiety (Yes/No) | 12206/2112 | 20/5 | Chi2[1]=0.209, p=0.648 |
| Number of things worried about during worst period of anxiety (One thing/More than one thing) | 6817/7627 | 11/14 | Chi2[1]=0.014, p=0.905 |
| Difficulty stopping worrying during worst period of anxiety (Yes/No) | 13324/1106 | 23/1 | Chi2[1]=0.067, p=0.795 |
| Multiple worries during worst period of anxiety (Yes/No) | 10190/4113 | 14/9 | Chi2[1]=0.753, p=0.386 |
| Tense, sore or aching muscles during worse period of anxiety (Yes/No) | 4237/9405 | 4/19 | Chi2[1]=1.416, p=0.234 |
| Difficulty concentrating during worst period of anxiety (Yes/No) | 10683/3636 | 18/6 | Chi2[1]=0.000, p>0.999 |
| More irritable than usual during worst period of anxiety (Yes/No) | 10080/3729 | 18/7 | Chi2[1]=0.000, p>0.999 |
| Restless during period of worst anxiety (Yes/No) | 8685/5282 | 13/9 | Chi2[1]=0.006, p=0.937 |
| Keyed up or on edge during worst period of anxiety (Yes/No) | 11054/3167 | 18/6 | Chi2[1]=0.006, p=0.940 |
| Frequent trouble falling or staying asleep during worst period of anxiety (Yes/No) | 10971/3507 | 17/8 | Chi2[1]=0.453, p=0.501 |
| Easily tired during worst period of anxiety (Yes/No) | 9060/4836 | 15/10 | Chi2[1]=0.112, p=0.738 |
| Professional informed about anxiety (Yes/No) | 8467/6331 | 15/10 | Chi2[1]=0.006, p=0.937 |
| Substances taken for anxiety (unprescribed)(Yes/No) | 913/58505 | 0/129 | Chi2[1]=1.124, p=0.289 |
| Substances taken for anxiety (prescribed)(Yes/No) | 5815/53603 | 7/122 | Chi2[1]=2.302, p=0.129 |
| Substances taken for anxiety (drugs or alcohol) (Yes/No) | 3221/56197 | 4/125 | Chi2[1]=0.938, p=0.333 |
| Activities undertaken to treat anxiety (talking therapies)(Yes/No) | 5559/53859 | 8/121 | Chi2[1]=1.162, p=0.281 |
| Activities undertaken to treat anxiety (other therapeutic activities e.g. yoga)(Yes/No) | 1673/57745 | 4/125 | p=0.786 |
| *Multiple answer questions* |  |  |  |
| Recent easy annoyance or irritability (1: not at all-4:nearly every day) | 1.31±0.002 | 1.29±0.051 | U=3731038, p=0.578 |
| Recent feelings of nervousness or anxiety (1: not at all-4:nearly every day) | 1.28±0.002 | 1.24±0.048 | U=3712823, p=0.457 |
| Recent inability to stop or control worrying (1: not at all-4:nearly every day) | 1.23±0.002 | 1.19±0.046 | U=3667636, p=0.244 |
| Recent feelings of foreboding (1: not at all-4:nearly every day) | 1.17±0.002 | 1.17±0.046 | U=3759084, p=0.797 |
| Recent trouble relaxing (1: not at all-4:nearly every day) | 1.31±0.003 | 1.28±0.053 | U=3748927, p=0.611 |
| Recent restlessness (1: not at all-4:nearly every day) | 1.14±0.002 | 1.16±0.044 | U=3727713, p=0.525 |
| Recent worrying too much about different things (1: not at all-4:nearly every day) | 1.31±0.003 | 1.26±0.052 | U=3610745, p=0.156 |
| Longest period spent worried or anxious (months) | 158.7±3.102 | 146.84±75.489 | U=110794.5, p=0.853 |
| Frequency of inability to stop worrying during worst period of anxiety (0:Never-3:Often) | 2.19±0.006 | 2.04±0.168 | U=166426, p=0.364 |
| Frequency of difficulty controlling worry during worst period of anxiety (0:Never-3:Often) | 2.23±0.006 | 2.12±0.156 | U=170080, p=0.471 |
| Impact on normal roles during worst period of anxiety (0: Not at all-3: A lot) | 1.89±0.008 | 2.04±0.168 | U=168953, p=0.416 |
| **Addictions** |  |  |  |
| Ever addicted to any substance or behaviour (Yes/No) | 4101/54604 | 13/113 | Chi2[1]=1.664, p=0.197 |
| Ever addicted to alcohol (Yes/No) | 1763/2037 | 6/6 | Chi2[1]=0.000, p>0.999 |
| Ongoing addiction to alcohol (Yes/No) | 757/981 | 3/3 | p>0.999 |
| Ever physically dependent on alcohol (Yes/No) | 553/1138 | 0/6 | p=0.186 |
| Ever addicted to prescription or over-the-counter medication (Yes/No) | 463/3589 | 3/10 | p=0.180 |
| Ever addicted to illicit or recreational drugs (Yes/No) | 357/3684 | 1/12 | p>0.999 |
| Ever addicted to a behaviour or miscellaneous | 1187/2842 | 5/8 | p=0.544 |
| Ongoing behavioural or miscellaneous addiction (Yes/No) | 533/633 | 1/3 | p=0.630 |
| **Alcohol use** |  |  |  |
| Frequency of drinking alcohol (0: Never-4: 4 or more times a week) | 2.87±0.005 | 2.92±0.107 | U=3675665, p=0.418 |
| Amount of alcohol drunk on a typical drinking day (1: 1-2 units-5: 10 or more units) | 2.11±0.005 | 2.23±0.112 | U=3258867, p=0.315 |
| Frequency of consuming 6 or more units of alcohol (1: Never-5: daily or almost daily) | 2.29±0.005 | 2.42±0.124 | U=3289252, p=0.397 |
| Frequency of inability to cease drinking in last year (1: Never-5: daily or almost daily) | 1.18±0.003 | 1.24±0.077 | U=1675941, p=0.267 |
| Frequency of failure to fulfil normal expectations due to drinking alcohol in last year (1: Never-5: daily or almost daily) | 1.09±0.002 | 1.12±0.045 | U=1728515, p=0.821 |
| Frequency of needing morning drink of alcohol after heavy drinking session in last year (1: Never-5: daily or almost daily) | 1.01±0.001 | 1.02±0.023 | U=1732080, p=0.790 |
| Frequency of feeling guilt or remorse after drinking alcohol in last year (1: Never-5: daily or almost daily) | 1.25±0.003 | 1.27±0.058 | U=1660273, p=0.271 |
| Frequency of memory loss due to drinking alcohol in the last year (1: Never-5: daily or almost daily) | 1.18±0.003 | 1.22±0.075 | U=1724728, p=0.864 |
| Ever been injured or injured someone else through drinking alcohol (0: No, 1:Yes, but not in last year, 2:Yes, during the last year) | 0.07±0.001 | 0.05±0.023 | U=3779575, p=0.573 |
| Ever had known person concerned about, or recommend reduction of, alcohol consumption (0: No, 1:Yes, but not in last year, 2:Yes, during the last year) | 0.20±0.002 | 0.19±0.048 | U=3790358, p=0.778 |
| Age when known person last commented about drinking habits (yrs) | 51.25±0.221 | 54.00±5.621 | U=9450, p=0.548 |
| **Cannabis use** |  |  |  |
| Ever taken cannabis (0:No, 1:1-2 times, 2:3-10 times, 3:11-100 times, 4: more than 100 times) | 0.52±0.004 | 0.60±0.101 | U=3741113, p=0.568 |
| Maximum frequency of taking cannabis (1:Less than once a month, 2:once a month or more but not every week, 3:once a week or more, but not every day, 4:Every day) | 1.70±0.008 | 1.76±0.179 | U=235986, p=0.657 |
| Age when last took cannabis (yrs) | 33.13±0.110 | 34.50±2.361 | U=237543, p=0.542 |
| **Unusual and psychotic experiences** |  |  |  |
| *Dual answer questions* |  |  |  |
| Ever seen an unreal vision (Yes/No) | 1587/57211 | 2/127 | p=0.590 |
| Ever heard an unreal voice (Yes/No) | 926/58200 | 1/128 | p=0.727 |
| Ever believed in unreal communications or signs (Yes/No) | 412/58815 | 0/129 | p>0.999 |
| Ever believed in unreal conspiracy against self (Yes/No) | 556/58676 | 1/128 | p>0.999 |
| Ever talked to a health professional about unusual or psychotic experiences (Yes/No) | 614/1996 | 0/3 | p>0.999 |
| Ever prescribed medication for unusual or psychotic experiences (Yes/No) | 341/2264 | 0/3 | p>0.999 |
| *Multiple answer questions* |  |  |  |
| Number of times seen an unreal vision | 33.79±1.908 | 1.00±0.000 | U=237, p=0.542 |
| Number of times heard an unreal voice | 5.08±0.568 | 1.00±0.000 | U=253, p=0.611 |
| Number of times believed in unreal communications or signs | 75.89±5.413 | - | N/A |
| Number of times believed in unreal conspiracy against self | 30.17±1.772 | 1.00±0.000 | U=167, p=0.798 |
| Frequency of unusual or psychotic experiences in last year (0:Not at all-4: Nearly every day or daily) | 0.55±0.020 | 0.00±0.000 | U=2729, p=0.495 |
| Age when first had unusual or psychotic experience (yrs) | 35.89±0.457 | 23.33±1.202 | U=2391, p=0.411 |
| Distress caused by unusual or psychotic experiences (0:Not distressing at all-4: Very distressing) | 1.47±0.026 | 2.00±1.000 | U=3044, p=0.530 |
| **Traumatic events** |  |  |  |
| Felt loved as a child (0:Never true-4: Very often true) | 3.23±0.004 | 3.26±0.088 | U=3668832, p=0.406 |
| Someone to take to doctor when needed as a child (0:Never true-4: Very often true) | 3.76±0.003 | 3.81±0.055 | U=3688593, p=0.306 |
| Been in a confiding relationship as an adult (0:Never true-4: Very often true) | 3.00±0.005 | 2.92±0.120 | U=3580736, p=0.611 |
| Able to pay rent/mortgage as an adult (0:Never true-4: Very often true) | 3.74±0.003 | 3.73±0.083 | U=3646507, p=0.354 |
| Been in serious accident believed to be life-threatening (0:Never, 1:Yes, but not in last 12 months, 2:Yes, within the last 12 months) | 0.14±0.001 | 0.11±0.028 | U=3709263, p=0.489 |
| Been involved in combat or exposed to war zone (0:Never, 1:Yes, but not in last 12 months, 2:Yes, within the last 12 months) | 0.06±0.001 | 0.08±0.024 | U=3725943, p=0.452 |
| Diagnosed with life-threatening illness (0:Never, 1:Yes, but not in last 12 months, 2:Yes, within the last 12 months) | 0.21±0.002 | 0.28±0.045 | U=3563954, p=0.049 |
| Victim of physically violent crime (0:Never, 1:Yes, but not in last 12 months, 2:Yes, within the last 12 months) | 0.25±0.002 | 0.24±0.038 | U=3802508, p=0.880 |
| Witnessed sudden violent death (0:Never, 1:Yes, but not in last 12 months, 2:Yes, within the last 12 months) | 0.20±0.002 | 0.19±0.037 | U=3804301, p=0.893 |
| Avoided activities or situations because of previous stressful experience in past month (0:Not at all-4:Extremely) | 0.23±0.002 | 0.16±0.046 | U=3588565, p=0.058 |
| Repeated disturbing thoughts of stressful experience in past month (0:Not at all-4:Extremely) | 0.31±0.003 | 0.25±0.044 | U=3756565, p=0.619 |
| Felt very upset when reminded of stressful experience in past month (0:Not at all-4:Extremely) | 0.38±0.003 | 0.33±0.056 | U=3719992, p=0.486 |
| Felt irritable or had angry outbursts in past month (0:Not at all-4:Extremely) | 0.57±0.005 | 0.57±0.114 | U=457833, p=0.881 |
| Felt distant from other people in past month (0:Not at all-4:Extremely) | 0.64±0.006 | 0.60±0.132 | U=454723, p=0.805 |
| **Happiness and subjective wellbeing** |  |  |  |
| General happiness (1:Extremely happy-6:Extremely unhappy) | 2.41±0.003 | 2.27±0.066 | U=3370877, p=0.019 |
| General happiness with own health (1:Extremely happy-6:Extremely unhappy) | 2.63±0.004 | 2.53±0.072 | U=3599723, p=0.216 |
| Belief that own life is meaningful (1:Not at all-5:an extreme amount) | 3.69±0.003 | 3.82±0.067 | U=3270667, p=0.095 |

**Supplementary Table 9.** Mental Health Questionnaire (MHQ) responses in female duplication carriers and female controls.

| **Depression** |  |  |  |
| --- | --- | --- | --- |
| *Dual answer questions* | **Female control** | **Female duplication** | **Statistical analysis** |
| Ever had prolonged feelings of sadness or depression? (Yes/No) | 47961/28074 | 161/107 | Chi2[1]=0.909, p=0.340 |
| Ever had prolonged loss of interest in normal activities? (Yes/No) | 34555/41439 | 119/150 | Chi2[1]=0.118, p=0.731 |
| Depression possibly related to stressful or traumatic event (Yes/No) | 38260/10514 | 127/37 | Chi2[1]=0.047, p=0.828 |
| Feelings of tiredness during worst episode of depression (Yes/No) | 37601/6389 | 132/20 | Chi2[1]=0.131, p=0.717 |
| Did your sleep change? (Yes/No) | 34858/7029 | 108/35 | Chi2[1]=5.497, p=0.019 |
| Trouble falling asleep (Yes/No) | 26427/8431 | 80/28 | Chi2[1]=0.095, p=0.757 |
| Sleeping too much (Yes/No) | 7294/27564 | 26/82 | Chi2[1]=0.469, p=0.494 |
| Waking too early (Yes/No) | 26554/8304 | 76/32 | Chi2[1]=1.693, p=0.193 |
| Difficulty concentrating during worst depression (Yes/No) | 34515/8185 | 105/41 | Chi2[1]=6.889, p=0.009 |
| Feelings of worthlessness during worst episode of depression (Yes/No) | 23677/21780 | 89/70 | Chi2[1]=0.810, p=0.368 |
| Thoughts of death during worst depression (Yes/No) | 25357/20529 | 97/60 | Chi2[1]=2.435, p=0.119 |
| Depression possibly related to childbirth (Yes/No) | 5579/38329 | 17/126 | Chi2[1]=0.028, p=0.867 |
| Professional informed about depression (Yes/No) | 33562/15162 | 103/60 | Chi2[1]=2.196, p=0.138 |
| Substances taken for depression (unprescribed)(Yes/No) | 3422/72843 | 15/254 | Chi2[1]=0.509, p=0.475 |
| Substances taken for depression (prescribed)(Yes/No) | 22586/53679 | 58/211 | Chi2[1]=7.964, p=0.005 |
| Substances taken for depression (drugs or alcohol)(Yes/No) | 5108/71157 | 28/241 | Chi2[1]=5.319, p=0.021 |
| Talking therapies (Yes/No) | 20576/55689 | 75/194 | Chi2[1]=0.070, p=0.792 |
| Other non-drug therapies (e.g. yoga) (Yes/No) | 8057/68208 | 31/238 | Chi2[1]=0.170, p=0.681 |
| *Multiple answer questions* |  |  |  |
| Trouble falling or staying asleep, or sleeping too much (1: not at all-4:nearly every day) | 1.82±0.003 | 1.84±0.059 | U=10142220, p=0.959 |
| Recent feelings of inadequacy (1: not at all-4:nearly every day) | 1.29±0.002 | 1.36±0.043 | U=9748556, p=0.080 |
| Recent trouble concentrating on things (1: not at all-4:nearly every day) | 1.24±0.002 | 1.28±0.057 | U=10141532, p=0.686 |
| Recent feelings of depression (1: not at all-4:nearly every day) | 1.30±0.002 | 1.32±0.041 | U=10207251, p=0.984 |
| Recent poor appetite or overeating (1: not at all-4:nearly every day) | 1.32±0.002 | 1.29±0.039 | U=10117010, p=0.752 |
| Recent thoughts of suicide or self-harm (1: not at all-4:nearly every day) | 1.05±0.001 | 1.08±0.021 | U=9863292, p=0.055 |
| Recent lack of interest or pleasure in doing things (1: not at all-4:nearly every day) | 1.25±0.002 | 1.26±0.037 | U=10213624, p=0.969 |
| Recent changes in speed/amount of moving or speaking (1: not at all-4:nearly every day) | 1.08±0.001 | 1.09±0.025 | U=10179958, p=0.723 |
| Recent feelings of tiredness or low energy (1: not at all-4:nearly every day) | 1.72±0.003 | 1.74±0.051 | U=10106470, p=0.700 |
| Fraction of day affected during worst episode of depression (1:less than half the day-4:all day long) | 2.91±0.004 | 2.88±0.067 | U=3539718, p=0.384 |
| Frequency of depressed days during worst episode of depression (1:less often-3:every day) | 2.34±0.003 | 2.28±0.051 | U=3595826, p=0.245 |
| Duration of worst depression (1:less than a month-6:over two years) | 3.14±0.007 | 3.21±0.124 | U=3773659, p=0.673 |
| Impact on normal roles during worst episode of depression (0:not at all-3:a lot) | 1.90±0.004 | 1.90±0.072 | U=3989873, p=0.936 |
| Age at first episode of depression (yrs) | 36.39±0.070 | 36.12±1.254 | U=34407443, p=0.741 |
| Age at last episode of depression (yrs) | 50.13±0.062 | 50.56±1.044 | U=3041125.5, p=0.534 |
| Lifetime number of depressed periods | 146.10±1.632 | 169.63±29.831 | U=3571729.5, p=0.778 |
| **Mania** |  |  |  |
| *Dual answer questions* | **Female control** | **Female duplication** | **Statistics** |
| Manifestations of mania (more talkative than usual) (Yes/No) | 3413/72852 | 10/259 | Chi2[1]=0.205, p=0.651 |
| Manifestations of mania (more restless than usual)(Yes/No) | 8154/68111 | 32/237 | Chi2[1]=0.291, p=0.590 |
| Manifestations of mania (my thoughts were racing)(Yes/No) | 6851/69414 | 26/243 | Chi2[1]=0.081, p=0.777 |
| Manifestations of mania (needed less sleep than usual)(Yes/No) | 2443/73822 | 6/263 | Chi2[1]=0.535, p=0.464 |
| Manifestations of mania (more creative than usual)(Yes/No) | 1629/74636 | 3/266 | Chi2[1]=0.894, p=0.344 |
| Manifestations of mania (was easily distracted)(Yes/No) | 6038/70227 | 21/248 | Chi2[1]=0.000, p>0.999 |
| Manifestations of mania (was more confident than usual)(Yes/No) | 1784/74481 | 6/263 | Chi2[1]=0.000, p>0.999 |
| Manifestations of mania (was more active than usual)(Yes/No) | 3555/72710 | 11/258 | Chi2[1]=0.090, p=0.765 |
| Ever had a period of mania/excitability? (Yes/No) | 2698/72121 | 9/251 | Chi2[1]=0.000, p>0.999 |
| Ever had a period of extreme irritability? (Yes/No) | 19657/54023 | 79/180 | Chi2[1]=1.737, p=0.187 |
| Severity of problems due to mania or irritability (No problems/caused problems) | 4881/12357 | 19/47 | Chi2[1]=0.000, p>0.999 |
| *Multiple answer questions* |  |  |  |
| Longest period of mania or irritability (1: less than 24hrs-3: a week or more) | 1.87±0.006 | 1.72±0.099 | U=552684, p=0.090 |
| **Anxiety** |  |  |  |
| *Dual answer questions* |  |  |  |
| Ever felt worried, tense or anxious for most of a month or longer (Yes/No) | 21624/48543 | 68/179 | Chi2[1]=1.099, p=0.295 |
| Ever worried more than most people would in similar situation (Yes/No) | 18504/44716 | 67/153 | Chi2[1]=0.097, p=0.755 |
| Stronger worrying (than other people) during period of worst anxiety (Yes/No) | 14985/3470 | 53/8 | Chi2[1]=0.943, p=0.331 |
| Worried most days during period of worst anxiety (Yes/No) | 23417/2601 | 75/10 | Chi2[1]=0.131, p=0.718 |
| Number of things worried about during worst period of anxiety (One thing/More than one thing) | 11014/15218 | 30/53 | Chi2[1]=0.014, p=0.905 |
| Difficulty stopping worrying during worst period of anxiety (Yes/No) | 24980/1270 | 84/3 | Chi2[1]=0.125, p=0.724 |
| Multiple worries during worst period of anxiety (Yes/No) | 20110/5702 | 65/20 | Chi2[1]=0.035, p=0.851 |
| Tense, sore or aching muscles during worse period of anxiety (Yes/No) | 10699/13800 | 32/50 | Chi2[1]=0.541, p=0.462 |
| Difficulty concentrating during worst period of anxiety (Yes/No) | 19545/6063 | 63/17 | Chi2[1]=0.143, p=0.705 |
| More irritable than usual during worst period of anxiety (Yes/No) | 17394/7065 | 57/22 | Chi2[1]=0.006, p=0.937 |
| Restless during period of worst anxiety (Yes/No) | 14700/10105 | 45/33 | Chi2[1]=0.028, p=0.868 |
| Keyed up or on edge during worst period of anxiety (Yes/No) | 20686/4962 | 71/11 | Chi2[1]=1.484, p=0.223 |
| Frequent trouble falling or staying asleep during worst period of anxiety (Yes/No) | 22437/3850 | 74/12 | Chi2[1]=0.001, p=0.977 |
| Easily tired during worst period of anxiety (Yes/No) | 19058/6138 | 53/28 | Chi2[1]=4.024, p=0.045 |
| Professional informed about anxiety (Yes/No) | 16937/9883 | 61/26 | Chi2[1]=1.521, p=0.217 |
| Substances taken for anxiety (unprescribed)(Yes/No) | 2502/73763 | 6/263 | Chi2[1]=0.631, p=0.427 |
| Substances taken for anxiety (prescribed)(Yes/No) | 11486/64779 | 38/231 | Chi2[1]=0.117, p=0.732 |
| Substances taken for anxiety (drugs or alcohol) (Yes/No) | 3946/72319 | 22/247 | Chi2[1]=4.330, p=0.037 |
| Activities undertaken to treat anxiety (talking therapies)(Yes/No) | 11568/64697 | 44/225 | Chi2[1]=0.209, p=0.647 |
| Activities undertaken to treat anxiety (other therapeutic activities e.g. yoga)(Yes/No) | 5824/70441 | 22/247 | Chi2[1]=0.048, p=0.827 |
| *Multiple answer questions* |  |  |  |
| Recent easy annoyance or irritability (1: not at all-4:nearly every day) | 1.34±0.002 | 1.34±0.037 | U=10190361, p=0.974 |
| Recent feelings of nervousness or anxiety (1: not at all-4:nearly every day) | 1.41±0.002 | 1.42±0.041 | U=10046119, p=0.583 |
| Recent inability to stop or control worrying (1: not at all-4:nearly every day) | 1.37±0.002 | 1.39±0.044 | U=10122194, p=0.872 |
| Recent feelings of foreboding (1: not at all-4:nearly every day) | 1.26±0.002 | 1.25±0.038 | U=9934211, p=0.368 |
| Recent trouble relaxing (1: not at all-4:nearly every day) | 1.43±0.003 | 1.42±0.043 | U=10143808, p=0.903 |
| Recent restlessness (1: not at all-4:nearly every day) | 1.17±0.002 | 1.19±0.034 | U=10179589, p=0.821 |
| Recent worrying too much about different things (1: not at all-4:nearly every day) | 1.47±0.003 | 1.46±0.044 | U=10036105, p=0.670 |
| Longest period spent worried or anxious (months) | 190.18±2.494 | 187.49±44.066 | U=727853.5, p=0.886 |
| Frequency of inability to stop worrying during worst period of anxiety (0:Never-3:Often) | 2.32±0.004 | 2.34±0.071 | U=1147891, p=0.824 |
| Frequency of difficulty controlling worry during worst period of anxiety (0:Never-3:Often) | 2.36±0.004 | 2.28±0.077 | U=1116642, p=0.396 |
| Impact on normal roles during worst period of anxiety (0: Not at all-3: A lot) | 1.84±0.006 | 1.77±0.098 | U=1136857, p=0.493 |
| **Addictions** |  |  |  |
| Ever addicted to any substance or behaviour (Yes/No) | 3810/71665 | 13/255 | Chi2[1]=0.000, p=0.994 |
| Ever addicted to alcohol (Yes/No) | 1337/2207 | 5/7 | p=0.773 |
| Ongoing addiction to alcohol (Yes/No) | 578/739 | 2/3 | p>0.999 |
| Ever physically dependent on alcohol (Yes/No) | 278/995 | 1/4 | p>0.999 |
| Ever addicted to prescription or over-the-counter medication (Yes/No) | 669/3085 | 2/10 | p>0.999 |
| Ever addicted to illicit or recreational drugs (Yes/No) | 217/3569 | 0/13 | p>0.999 |
| Ever addicted to a behaviour or miscellaneous | 642/3136 | 2/11 | p>0.999 |
| Ongoing behavioural or miscellaneous addiction (Yes/No) | 327/301 | 1/1 | p>0.999 |
| **Alcohol use** |  |  |  |
| Frequency of drinking alcohol (0: Never-4: 4 or more times a week) | 2.43±0.005 | 2.53±0.077 | U=9813035, p=0.220 |
| Amount of alcohol drunk on a typical drinking day (1: 1-2 units-5: 10 or more units) | 1.60±0.003 | 1.64±0.059 | U=8307641, p=0.593 |
| Frequency of consuming 6 or more units of alcohol (1: Never-5: daily or almost daily) | 1.69±0.004 | 1.73±0.069 | U=8397984, p=0.681 |
| Frequency of inability to cease drinking in last year (1: Never-5: daily or almost daily) | 1.21±0.004 | 1.22±0.052 | U=2001799, p=0.271 |
| Frequency of failure to fulfil normal expectations due to drinking alcohol in last year (1: Never-5: daily or almost daily) | 1.08±0.002 | 1.07±0.024 | U=2080079, p=0.856 |
| Frequency of needing morning drink of alcohol after heavy drinking session in last year (1: Never-5: daily or almost daily) | 1.01±0.001 | 1.01±0.008 | U=2083277, p=0.340 |
| Frequency of feeling guilt or remorse after drinking alcohol in last year (1: Never-5: daily or almost daily) | 1.33±0.004 | 1.41±0.074 | U=2000228, p=0.260 |
| Frequency of memory loss due to drinking alcohol in the last year (1: Never-5: daily or almost daily) | 1.17±0.003 | 1.21±0.047 | U=2012567, p=0.227 |
| Ever been injured or injured someone else through drinking alcohol (0: No, 1:Yes, but not in last year, 2:Yes, during the last year) | 0.03±0.001 | 0.05±0.015 | U=10126266, p=0.232 |
| Ever had known person concerned about, or recommend reduction of, alcohol consumption (0: No, 1:Yes, but not in last year, 2:Yes, during the last year) | 0.07±0.001 | 0.09±0.023 | U=10136720, p=0.435 |
| Age when known person last commented about drinking habits (yrs) | 50.08±0.292 | 47.00±4.668 | U=6313, p=0.549 |
| **Cannabis use** |  |  |  |
| Ever taken cannabis (0:No, 1:1-2 times, 2:3-10 times, 3:11-100 times, 4: more than 100 times) | 0.35±0.003 | 0.31±0.048 | U=10142276, p=0.678 |
| Maximum frequency of taking cannabis (1:Less than once a month, 2:once a month or more but not every week, 3:once a week or more, but not every day, 4:Every day) | 1.57±0.008 | 1.43±0.127 | U=288177, p=0.261 |
| Age when last took cannabis (yrs) | 31.14±0.105 | 31.81±1.930 | U=331052.5, p=0.732 |
| **Unusual and psychotic experiences** |  |  |  |
| *Dual answer questions* |  |  |  |
| Ever seen an unreal vision (Yes/No) | 2692/72758 | 4/262 | Chi2[1]=2.2715, p=0.099 |
| Ever heard an unreal voice (Yes/No) | 1384/74525 | 2/264 | p=0.251 |
| Ever believed in unreal communications or signs (Yes/No) | 531/75514 | 1/267 | p>0.999 |
| Ever believed in unreal conspiracy against self (Yes/No) | 498/75610 | 1/267 | p>0.999 |
| Ever talked to a health professional about unusual or psychotic experiences (Yes/No) | 777/3186 | 2/6 | p=660 |
| Ever prescribed medication for unusual or psychotic experiences (Yes/No) | 390/3568 | 0/8 | p>0.999 |
| *Multiple answer questions* |  |  |  |
| Number of times seen an unreal vision | 31.80±1.424 | 65.33±62.336 | U=2047, p=0.255 |
| Number of times heard an unreal voice | 3.14±0.153 | 3.00±0.000 | U=485, p=0.326 |
| Number of times believed in unreal communications or signs | 62.82±4.532 | - | N/A |
| Number of times believed in unreal conspiracy against self | 26.14±1.764 | 1.00±0.000 | U=195, p>0.999 |
| Frequency of unusual or psychotic experiences in last year (0:Not at all-4: Nearly every day or daily) | 0.42±0.013 | 0.88±0.479 | U=13542, p=0.412 |
| Age when first had unusual or psychotic experience (yrs) | 34.75±0.355 | 35.00±2.887 | U=4632.5, p=0.760 |
| Distress caused by unusual or psychotic experiences (0:Not distressing at all-4: Very distressing) | 1.41±0.022 | 1.38±0.420 | U=15121, p=0.888 |
| **Traumatic events** |  |  |  |
| Felt loved as a child (0:Never true-4: Very often true) | 3.24±0.004 | 3.22±0.056 | U=9878411, p=0.419 |
| Someone to take to doctor when needed as a child (0:Never true-4: Very often true) | 3.73±0.003 | 3.75±0.037 | U=9967646, p=0.664 |
| Been in a confiding relationship as an adult (0:Never true-4: Very often true) | 2.98±0.005 | 2.95±0.078 | U=9601257, p=0.538 |
| Able to pay rent/mortgage as an adult (0:Never true-4: Very often true) | 3.70±0.003 | 3.67±0.053 | U=9729754, p=0.415 |
| Been in serious accident believed to be life-threatening (0:Never, 1:Yes, but not in last 12 months, 2:Yes, within the last 12 months) | 0.07±0.001 | 0.10±0.019 | U=9968957, p=0.076 |
| Been involved in combat or exposed to war zone (0:Never, 1:Yes, but not in last 12 months, 2:Yes, within the last 12 months) | 0.01±0.000 | 0.01±0.005 | U=10188297, p= 0.447 |
| Diagnosed with life-threatening illness (0:Never, 1:Yes, but not in last 12 months, 2:Yes, within the last 12 months) | 0.17±0.002 | 0.15±0.025 | U=10010593, p=0.481 |
| Victim of physically violent crime (0:Never, 1:Yes, but not in last 12 months, 2:Yes, within the last 12 months) | 0.14±0.001 | 0.13±0.021 | U=10145265, p=0.709 |
| Witnessed sudden violent death (0:Never, 1:Yes, but not in last 12 months, 2:Yes, within the last 12 months) | 0.09±0.001 | 0.10±0.020 | U=10105320, p=0.612 |
| Avoided activities or situations because of previous stressful experience in past month (0:Not at all-4:Extremely) | 0.34±0.003 | 0.35±0.045 | U=10044896, p=0.565 |
| Repeated disturbing thoughts of stressful experience in past month (0:Not at all-4:Extremely) | 0.44±0.003 | 0.48±0.054 | U=10171376, p=0.918 |
| Felt very upset when reminded of stressful experience in past month (0:Not at all-4:Extremely) | 0.62±0.003 | 0.60±0.054 | U=10004012, p=0.543 |
| Felt irritable or had angry outbursts in past month (0:Not at all-4:Extremely) | 0.49±0.004 | 0.52±0.069 | U=2594194, p=0.893 |
| Felt distant from other people in past month (0:Not at all-4:Extremely) | 0.64±0.005 | 0.75±0.086 | U=2426849, p=0.205 |
| **Happiness and subjective wellbeing** |  |  |  |
| General happiness (1:Extremely happy-6:Extremely unhappy) | 2.42±0.003 | 2.50±0.045 | U=9541782, p=0.130 |
| General happiness with own health (1:Extremely happy-6:Extremely unhappy) | 2.63±0.003 | 2.62±0.057 | U=10173007.5, p=0.968 |
| Belief that own life is meaningful (1:Not at all-5:an extreme amount) | 3.70±0.003 | 3.65±0.049 | U=9384071, p=0.240 |

**Supplementary Table 10.** Prescribed medications used to treat GORD, blistering skin disorders and psychiatric symptoms in male duplication carriers and male controls.

| **Body system** | **Medication Classification** | **UK Biobank code** | **Drug name** | **Male controls prescribed** | **Male controls not prescribed** | **Male duplication carriers prescribed** | **Male duplication carriers not prescribed** | **P Value** |
| --- | --- | --- | --- | --- | --- | --- | --- | --- |
| Gastrointestinal | Antacid | 2038507816 | Aluminium hydroxide | 117 | 192709 | 1 | 413 | 0.224 |
|  |  | 1140865186 | Aluminium hydroxide product |  |  |  |  |  |
|  |  | 1140850920 | Aluminium hydroxide+belladonna mixture |  |  |  |  |  |
|  |  | 1140881320 | Magnesium carbonate |  |  |  |  |  |
|  |  | 1140877826 | Sodium bicarbonate |  |  |  |  |  |
|  |  | 1140881318 | Co-magaldrox |  |  |  |  |  |
|  |  | 1141157480 | Co-magaldrox product |  |  |  |  |  |
|  |  | 1140881324 | Magnesium trisilicate |  |  |  |  |  |
|  | Proton Pump Inhibitor | 1141177526 | Esomeprazole | 16051 | 176775 | 46 | 368 | Chi2[1]=3.845, p=0.050 |
|  |  | 1140864752 | Lansoprazole |  |  |  |  |  |
|  |  | 1140865634 | Omeprazole |  |  |  |  |  |
|  |  | 1140929012 | Pantoprazole |  |  |  |  |  |
|  |  | 1141168584 | Rabeprazole |  |  |  |  |  |
|  | H2 Receptor Antagonist | 1140865618 | Nizatidine | 2940 | 189886 | 2 | 412 | Chi2[1]=2.335, p=0.126 |
|  |  | 1140865608 | Famotidine |  |  |  |  |  |
|  |  | 1140865426 | Cimetidine |  |  |  |  |  |
|  |  | 1140879406 | Ranitidine |  |  |  |  |  |
|  |  |  |  |  |  |  |  |  |
| Skin | Blistering disorders | 1140869930 | Azathioprine | 378 | 192448 | 0 | 414 | >0.999 |
|  |  | 1140925978 | Mycophenolate |  |  |  |  |  |
|  |  | 1140921992 | Nicotinamide 4% topical gel |  |  |  |  |  |
|  |  | 1140870914 | Nicotinamide product |  |  |  |  |  |
|  |  | 1140874104 | Dapsone |  |  |  |  |  |
|  |  |  |  |  |  |  |  |  |
| Psychiatric | ADHD | 1140867894 | Pemoline | 9 | 192817 | 0 | 414 | >0.999 |
|  | ADHD | 1140917138 | Ritalin 10mg tablet |  |  |  |  |  |
|  | ADHD | 1141199446 | Atomoxetine |  |  |  |  |  |
|  | ADHD | 1141180976 | Dexamfetamine |  |  |  |  |  |
|  | ADHD | 1140879680 | Dexamphetamine |  |  |  |  |  |
|  | Alcohol | 1140872484 | Antabuse 200mg tablet | 52 | 192774 | 1 | 413 | 0.107 |
|  | Alcohol | 1140872480 | Disulfiram |  |  |  |  |  |
|  | Alcohol | 1140863350 | Librium 5mg tablet |  |  |  |  |  |
|  | Alcohol | 1140863328 | Chlordiazepoxide |  |  |  |  |  |
|  | Alcohol | 1140863034 | Chlormethiazole |  |  |  |  |  |
|  | Alcohol | 1140909798 | Clomethiazole |  |  |  |  |  |
|  | Alcohol | 1140926990 | Acamprosate calcium |  |  |  |  |  |
|  | Alcohol | 1140926994 | Campral ec 333mg e/c tablet |  |  |  |  |  |
|  | Alcohol | 1141157338 | Disulfiram product |  |  |  |  |  |
|  | Antidepressant | 1140867938 | Amitriptyline+chlordiazepoxide 12.5mg/5mg capsule | 5376 | 187450 | 6 | 408 | Chi2[1]=2.262, p=0.133 |
|  | Antidepressant | 1140867920 | Moclobemide |  |  |  |  |  |
|  | Antidepressant | 1140867914 | Tranylcypromine |  |  |  |  |  |
|  | Antidepressant | 1140867888 | Paroxetine |  |  |  |  |  |
|  | Antidepressant | 1140867878 | Sertraline |  |  |  |  |  |
|  | Antidepressant | 1140867856 | Isocarboxazid |  |  |  |  |  |
|  | Antidepressant | 1140867852 | Nardil 15mg tablet |  |  |  |  |  |
|  | Antidepressant | 1140867850 | Phenelzine |  |  |  |  |  |
|  | Antidepressant | 1140867818 | Nortriptyline |  |  |  |  |  |
|  | Antidepressant | 1140867812 | Norval 10mg tablet |  |  |  |  |  |
|  | Antidepressant | 1140867774 | Amoxapine |  |  |  |  |  |
|  | Antidepressant | 1140867756 | Trimipramine |  |  |  |  |  |
|  | Antidepressant | 1140867726 | Lofepramine |  |  |  |  |  |
|  | Antidepressant | 1140867640 | Doxepin |  |  |  |  |  |
|  | Antidepressant | 1140867632 | Dothapax 25mg capsule |  |  |  |  |  |
|  | Antidepressant | 1140867624 | Prothiaden 25mg capsule |  |  |  |  |  |
|  | Antidepressant | 1140856074 | Butriptyline |  |  |  |  |  |
|  | Antidepressant | 1140909806 | Dosulepin |  |  |  |  |  |
|  | Antidepressant | 1140910504 | MAOI - isocarboxazid |  |  |  |  |  |
|  | Antidepressant | 1140910704 | MAOI - phenelzine |  |  |  |  |  |
|  | Antidepressant | 1140910820 | MAOI - tranylcypromine |  |  |  |  |  |
|  | Antidepressant | 1140916282 | Venlafaxine |  |  |  |  |  |
|  | Antidepressant | 1140917460 | Nefazodone |  |  |  |  |  |
|  | Antidepressant | 1140921600 | Citalopram |  |  |  |  |  |
|  | Antidepressant | 1141151978 | Reboxetine |  |  |  |  |  |
|  | Antidepressant | 1141152732 | Mirtazapine |  |  |  |  |  |
|  | Antidepressant | 1141176854 | Bupropion |  |  |  |  |  |
|  | Antidepressant | 1141180212 | Escitalopram |  |  |  |  |  |
|  | Antidepressant | 1141200564 | Duloxetine |  |  |  |  |  |
|  | Antidepressant | 1140882244 | Molipaxin 50mg capsule |  |  |  |  |  |
|  | Antidepressant | 1140879730 | Buspirone |  |  |  |  |  |
|  | Antidepressant | 1140879668 | Selegiline |  |  |  |  |  |
|  | Antidepressant | 1140879634 | Trazodone |  |  |  |  |  |
|  | Antidepressant | 1140879632 | Protriptyline |  |  |  |  |  |
|  | Antidepressant | 1140879630 | Imipramine |  |  |  |  |  |
|  | Antidepressant | 1140879628 | Dothiepin |  |  |  |  |  |
|  | Antidepressant | 1140879624 | Desipramine |  |  |  |  |  |
|  | Antidepressant | 1140879620 | Clomipramine |  |  |  |  |  |
|  | Antidepressant | 1140879616 | Amitriptyline |  |  |  |  |  |
|  | Antidepressant | 1140879556 | Mianserin |  |  |  |  |  |
|  | Antidepressant | 1140879544 | Fluvoxamine |  |  |  |  |  |
|  | Antidepressant | 1140879540 | Fluoxetine |  |  |  |  |  |
|  | Antipsychotic | 1140868170 | Prochlorperazine | 742 | 192084 | 2 | 412 | 0.676 |
|  | Antipsychotic | 1140868120 | Trifluoperazine |  |  |  |  |  |
|  | Antipsychotic | 1140867572 | Piportil depot 50mg/1ml oily injection |  |  |  |  |  |
|  | Antipsychotic | 1140867456 | Modecate 12.5mg/0.5ml oily injection |  |  |  |  |  |
|  | Antipsychotic | 1140867444 | Risperidone |  |  |  |  |  |
|  | Antipsychotic | 1140867420 | Clozapine |  |  |  |  |  |
|  | Antipsychotic | 1140867414 | Loxapac 10mg capsule |  |  |  |  |  |
|  | Antipsychotic | 1140867406 | Loxapine |  |  |  |  |  |
|  | Antipsychotic | 1140867398 | Fluphenazine decanoate |  |  |  |  |  |
|  | Antipsychotic | 1140867332 | Trifluperidol |  |  |  |  |  |
|  | Antipsychotic | 1140867304 | Sulpiride |  |  |  |  |  |
|  | Antipsychotic | 1140867218 | Pimozide |  |  |  |  |  |
|  | Antipsychotic | 1140867208 | Perphenazine |  |  |  |  |  |
|  | Antipsychotic | 1140867184 | Haldol 5mg tablet |  |  |  |  |  |
|  | Antipsychotic | 1140867168 | Haloperidol |  |  |  |  |  |
|  | Antipsychotic | 1140867156 | Moditen 1mg tablet |  |  |  |  |  |
|  | Antipsychotic | 1140867150 | Flupenthixol |  |  |  |  |  |
|  | Antipsychotic | 1140867136 | Neulactil 2.5mg tablet |  |  |  |  |  |
|  | Antipsychotic | 1140867134 | Pericyazine |  |  |  |  |  |
|  | Antipsychotic | 1140867122 | Nozinan 25mg tablet |  |  |  |  |  |
|  | Antipsychotic | 1140867084 | Droperidol |  |  |  |  |  |
|  | Antipsychotic | 1140867078 | Benperidol |  |  |  |  |  |
|  | Antipsychotic | 1140863416 | Largactil 10mg tablet |  |  |  |  |  |
|  | Antipsychotic | 1140856052 | Chlorprothixene |  |  |  |  |  |
|  | Antipsychotic | 1140856004 | Moditen enanthate 25mg/ml injection |  |  |  |  |  |
|  | Antipsychotic | 1140909800 | Flupentixol |  |  |  |  |  |
|  | Antipsychotic | 1140909802 | Levomepromazine |  |  |  |  |  |
|  | Antipsychotic | 1140909804 | Pipotiazine |  |  |  |  |  |
|  | Antipsychotic | 1140928916 | Olanzapine |  |  |  |  |  |
|  | Antipsychotic | 1141152848 | Quetiapine |  |  |  |  |  |
|  | Antipsychotic | 1141153490 | Amisulpride |  |  |  |  |  |
|  | Antipsychotic | 1141177762 | Risperdal 0.5mg tablet |  |  |  |  |  |
|  | Antipsychotic | 1141195974 | Aripiprazole |  |  |  |  |  |
|  | Antipsychotic | 1141202024 | Abilify 5mg tablet |  |  |  |  |  |
|  | Antipsychotic | 1140882382 | Butyrophenone product |  |  |  |  |  |
|  | Antipsychotic | 1140879750 | Thioridazine |  |  |  |  |  |
|  | Antipsychotic | 1140879746 | Promazine |  |  |  |  |  |
|  | Antipsychotic | 1140879674 | Pipothiazine |  |  |  |  |  |
|  | Antipsychotic | 1140879658 | Chlorpromazine |  |  |  |  |  |
|  | Antipsychotic | 1140882100 | Zuclopenthixol |  |  |  |  |  |
|  | Antipsychotic | 1140882098 | Fluphenazine |  |  |  |  |  |
|  | Antipsychotic | 1140882320 | Clozaril 25mg tablet |  |  |  |  |  |
|  | Antipsychotic | 1141169714 | Zotepine |  |  |  |  |  |
|  | Anxiety | 1140872236 | Neurontin 100mg capsule | 1972 | 190854 | 5 | 409 | 0.621 |
|  | Anxiety | 1140872228 | Gabapentin |  |  |  |  |  |
|  | Anxiety | 1140872150 | Clonazepam |  |  |  |  |  |
|  | Anxiety | 1140863442 | Oxazepam |  |  |  |  |  |
|  | Anxiety | 1140863374 | Nobrium 5mg capsule |  |  |  |  |  |
|  | Anxiety | 1140863364 | Ativan 1mg tablet |  |  |  |  |  |
|  | Anxiety | 1140863308 | Alprazolam |  |  |  |  |  |
|  | Anxiety | 1140863302 | Lorazepam |  |  |  |  |  |
|  | Anxiety | 1140863268 | Clobazam |  |  |  |  |  |
|  | Anxiety | 1140863262 | Chlormezanone |  |  |  |  |  |
|  | Anxiety | 1140863250 | Valium 2mg/5ml syrup |  |  |  |  |  |
|  | Anxiety | 1140863244 | Valium 2mg tablet |  |  |  |  |  |
|  | Anxiety | 1140863210 | Normison 10mg capsule |  |  |  |  |  |
|  | Anxiety | 1140863202 | Temazepam |  |  |  |  |  |
|  | Anxiety | 1140863194 | Mogadon 5mg tablet |  |  |  |  |  |
|  | Anxiety | 1140863182 | Nitrazepam |  |  |  |  |  |
|  | Anxiety | 1140863176 | Lormetazepam |  |  |  |  |  |
|  | Anxiety | 1140863152 | Diazepam |  |  |  |  |  |
|  | Anxiety | 1140863120 | Loprazolam |  |  |  |  |  |
|  | Anxiety | 1140863110 | Flurazepam |  |  |  |  |  |
|  | Anxiety | 1140863022 | Noctec 500mg capsule |  |  |  |  |  |
|  | Anxiety | 1140855914 | Triazolam |  |  |  |  |  |
|  | Anxiety | 1140855856 | Valium 10mg suppository |  |  |  |  |  |
|  | Anxiety | 1141157496 | Diazepam product |  |  |  |  |  |
|  | Anxiety | 1141168436 | Tiagabine |  |  |  |  |  |
|  | Anxiety | 1141200004 | Pregabalin |  |  |  |  |  |
|  | Mixed antidepressant antipsychotic | 1140867948 | Amitriptyline hydrochloride+perphenazine 10mg/2mg tablet | 9 | 192817 | 0 | 414 | >0.999 |
|  | Mixed antidepressant antipsychotic | 1140867944 | Tranylcypromine+trifluoperazine 10mg/1mg tablet |  |  |  |  |  |
|  | Mixed antidepressant antipsychotic | 1140867942 | Fluphenazine hcl+nortriptyline 500micrograms/10mg tablet |  |  |  |  |  |
|  | Mixed antidepressant antipsychotic | 1140867940 | Fluphenazine hydrochloride+nortriptyline 1.5mg/30mg tablet |  |  |  |  |  |
|  | Mixed antidepressant antipsychotic | 1140867930 | Motival tablet |  |  |  |  |  |
|  | Mixed antidepressant antipsychotic | 1140882240 | Motipress tablet |  |  |  |  |  |
|  | Mood Stabiliser | 1140872302 | Lamictal 25mg tablet | 1381 | 191445 | 5 | 409 | 0.229 |
|  | Mood Stabiliser | 1140872290 | Lamotrigine |  |  |  |  |  |
|  | Mood Stabiliser | 1140872214 | Valproic acid |  |  |  |  |  |
|  | Mood Stabiliser | 1140872200 | Epilim 100mg crushable tablet |  |  |  |  |  |
|  | Mood Stabiliser | 1140872198 | Sodium valproate |  |  |  |  |  |
|  | Mood Stabiliser | 1140872072 | Tegretol 100mg tablet |  |  |  |  |  |
|  | Mood Stabiliser | 1140872064 | Carbamazepine product |  |  |  |  |  |
|  | Mood Stabiliser | 1140867520 | Li-liquid 5.4mmol/5ml oral solution |  |  |  |  |  |
|  | Mood Stabiliser | 1140867518 | Litarex 564mg m/r tablet |  |  |  |  |  |
|  | Mood Stabiliser | 1140867504 | Priadel 200mg m/r tablet |  |  |  |  |  |
|  | Mood Stabiliser | 1140867498 | Liskonum 450mg m/r tablet |  |  |  |  |  |
|  | Mood Stabiliser | 1140867494 | Camcolit 250 tablet |  |  |  |  |  |
|  | Mood Stabiliser | 1140867490 | Lithium product |  |  |  |  |  |
|  | Mood Stabiliser | 1140910976 | Lithonate 400mg m/r tablet |  |  |  |  |  |
|  | Mood Stabiliser | 1140917270 | Li-liquid 509mg/5ml oral solution |  |  |  |  |  |
|  | Mood Stabiliser | 1140923484 | Topiramate |  |  |  |  |  |
|  | Mood Stabiliser | 1141172838 | Depakote 250mg e/c tablet |  |  |  |  |  |
|  | Mood Stabiliser | 2038459704 | Carbamazepine |  |  |  |  |  |
|  | Side Effect | 1140865394 | Hyoscine butylbromide | 176 | 192650 | 0 | 414 | >0.999 |
|  | Side Effect | 1140883476 | Procyclidine |  |  |  |  |  |
|  | Side Effect | 1140883510 | Benzhexol |  |  |  |  |  |
|  | Side Effect | 1140883514 | Benztropine |  |  |  |  |  |
|  | Side Effect | 1140909816 | Trihexyphenidyl |  |  |  |  |  |
|  | Side Effect | 1140909818 | Benzatropine |  |  |  |  |  |
|  | Side Effect | 1141157482 | Hyoscine product |  |  |  |  |  |
|  | Side Effect | 1140882232 | Kwells tablet |  |  |  |  |  |
|  | Side Effect | 1140882104 | Hyoscine |  |  |  |  |  |

**Supplementary Table 11.** Prescribed medications used to treat GORD, blistering skin disorders and psychiatric symptoms in female duplication carriers and female controls.

| **Body system** | **Medication Classification** | **UK Biobank code** | **Drug name** | **Female controls prescribed** | **Female controls not prescribed** | **Female duplication carriers prescribed** | **Female duplication carriers not prescribed** | **P-value** |
| --- | --- | --- | --- | --- | --- | --- | --- | --- |
| Gastrointestinal | Antacid | 2038507816 | Aluminium hydroxide | 171 | 227064 | 1 | 937 | 0.508 |
|  |  | 1140865186 | Aluminium hydroxide product |  |  |  |  |  |
|  |  | 1140850920 | Aluminium hydroxide+belladonna mixture |  |  |  |  |  |
|  |  | 1140881320 | Magnesium carbonate |  |  |  |  |  |
|  |  | 1140877826 | Sodium bicarbonate |  |  |  |  |  |
|  |  | 1140881318 | Co-magaldrox |  |  |  |  |  |
|  |  | 1141157480 | Co-magaldrox product |  |  |  |  |  |
|  |  | 1140881324 | Magnesium trisilicate |  |  |  |  |  |
|  | Proton Pump Inhibitor | 1141177526 | Esomeprazole | 20353 | 206882 | 85 | 853 | Chi2[1]=0.003, p=0.956 |
|  |  | 1140864752 | Lansoprazole |  |  |  |  |  |
|  |  | 1140865634 | Omeprazole |  |  |  |  |  |
|  |  | 1140929012 | Pantoprazole |  |  |  |  |  |
|  |  | 1141168584 | Rabeprazole |  |  |  |  |  |
|  | H2 Receptor Antagonist | 1140865618 | Nizatidine | 3764 | 223471 | 15 | 923 | Chi2[1]<0.001, p=0.993 |
|  |  | 1140865608 | Famotidine |  |  |  |  |  |
|  |  | 1140865426 | Cimetidine |  |  |  |  |  |
|  |  | 1140879406 | Ranitidine |  |  |  |  |  |
|  |  |  |  |  |  |  |  |  |
| Skin | Blistering disorders | 1140869930 | Azathioprine | 348 | 226887 | 0 | 938 | 0.412 |
|  |  | 1140925978 | Mycophenolate |  |  |  |  |  |
|  |  | 1140921992 | Nicotinamide 4% topical gel |  |  |  |  |  |
|  |  | 1140870914 | Nicotinamide product |  |  |  |  |  |
|  |  | 1140874104 | Dapsone |  |  |  |  |  |
|  |  |  |  |  |  |  |  |  |
| Psychiatric | ADHD | 1140867894 | Pemoline | 7 | 227228 | 0 | 938 | >0.999 |
|  | ADHD | 1140917138 | Ritalin 10mg tablet |  |  |  |  |  |
|  | ADHD | 1141199446 | Atomoxetine |  |  |  |  |  |
|  | ADHD | 1141180976 | Dexamfetamine |  |  |  |  |  |
|  | ADHD | 1140879680 | Dexamphetamine |  |  |  |  |  |
|  | Alcohol | 1140872484 | Antabuse 200mg tablet | 26 | 227209 | 0 | 938 | >0.999 |
|  | Alcohol | 1140872480 | Disulfiram |  |  |  |  |  |
|  | Alcohol | 1140863350 | Librium 5mg tablet |  |  |  |  |  |
|  | Alcohol | 1140863328 | Chlordiazepoxide |  |  |  |  |  |
|  | Alcohol | 1140863034 | Chlormethiazole |  |  |  |  |  |
|  | Alcohol | 1140909798 | Clomethiazole |  |  |  |  |  |
|  | Alcohol | 1140926990 | Acamprosate calcium |  |  |  |  |  |
|  | Alcohol | 1140926994 | Campral ec 333mg e/c tablet |  |  |  |  |  |
|  | Alcohol | 1141157338 | Disulfiram product |  |  |  |  |  |
|  | Antidepressant | 1140867938 | Amitriptyline+chlordiazepoxide 12.5mg/5mg capsule | 10789 | 216446 | 44 | 894 | Chi2[1]<0.001, p=0.996 |
|  | Antidepressant | 1140867920 | moclobemide |  |  |  |  |  |
|  | Antidepressant | 1140867914 | Tranylcypromine |  |  |  |  |  |
|  | Antidepressant | 1140867888 | Paroxetine |  |  |  |  |  |
|  | Antidepressant | 1140867878 | Sertraline |  |  |  |  |  |
|  | Antidepressant | 1140867856 | Isocarboxazid |  |  |  |  |  |
|  | Antidepressant | 1140867852 | Nardil 15mg tablet |  |  |  |  |  |
|  | Antidepressant | 1140867850 | Phenelzine |  |  |  |  |  |
|  | Antidepressant | 1140867818 | Nortriptyline |  |  |  |  |  |
|  | Antidepressant | 1140867812 | Norval 10mg tablet |  |  |  |  |  |
|  | Antidepressant | 1140867774 | Amoxapine |  |  |  |  |  |
|  | Antidepressant | 1140867756 | Trimipramine |  |  |  |  |  |
|  | Antidepressant | 1140867726 | Lofepramine |  |  |  |  |  |
|  | Antidepressant | 1140867640 | Doxepin |  |  |  |  |  |
|  | Antidepressant | 1140867632 | Dothapax 25mg capsule |  |  |  |  |  |
|  | Antidepressant | 1140867624 | Prothiaden 25mg capsule |  |  |  |  |  |
|  | Antidepressant | 1140856074 | Butriptyline |  |  |  |  |  |
|  | Antidepressant | 1140909806 | Dosulepin |  |  |  |  |  |
|  | Antidepressant | 1140910504 | MAOI - isocarboxazid |  |  |  |  |  |
|  | Antidepressant | 1140910704 | MAOI - phenelzine |  |  |  |  |  |
|  | Antidepressant | 1140910820 | MAOI - tranylcypromine |  |  |  |  |  |
|  | Antidepressant | 1140916282 | Venlafaxine |  |  |  |  |  |
|  | Antidepressant | 1140917460 | Nefazodone |  |  |  |  |  |
|  | Antidepressant | 1140921600 | Citalopram |  |  |  |  |  |
|  | Antidepressant | 1141151978 | Reboxetine |  |  |  |  |  |
|  | Antidepressant | 1141152732 | Mirtazapine |  |  |  |  |  |
|  | Antidepressant | 1141176854 | Bupropion |  |  |  |  |  |
|  | Antidepressant | 1141180212 | Escitalopram |  |  |  |  |  |
|  | Antidepressant | 1141200564 | Duloxetine |  |  |  |  |  |
|  | Antidepressant | 1140882244 | Molipaxin 50mg capsule |  |  |  |  |  |
|  | Antidepressant | 1140879730 | Buspirone |  |  |  |  |  |
|  | Antidepressant | 1140879668 | Selegiline |  |  |  |  |  |
|  | Antidepressant | 1140879634 | Trazodone |  |  |  |  |  |
|  | Antidepressant | 1140879632 | Protriptyline |  |  |  |  |  |
|  | Antidepressant | 1140879630 | Imipramine |  |  |  |  |  |
|  | Antidepressant | 1140879628 | Dothiepin |  |  |  |  |  |
|  | Antidepressant | 1140879624 | Desipramine |  |  |  |  |  |
|  | Antidepressant | 1140879620 | Clomipramine |  |  |  |  |  |
|  | Antidepressant | 1140879616 | Amitriptyline |  |  |  |  |  |
|  | Antidepressant | 1140879556 | Mianserin |  |  |  |  |  |
|  | Antidepressant | 1140879544 | Fluvoxamine |  |  |  |  |  |
|  | Antidepressant | 1140879540 | Fluoxetine |  |  |  |  |  |
|  | Antipsychotic | 1140868170 | Prochlorperazine | 1033 | 226202 | 5 | 933 | 0.624 |
|  | Antipsychotic | 1140868120 | Trifluoperazine |  |  |  |  |  |
|  | Antipsychotic | 1140867572 | Piportil depot 50mg/1ml oily injection |  |  |  |  |  |
|  | Antipsychotic | 1140867456 | Modecate 12.5mg/0.5ml oily injection |  |  |  |  |  |
|  | Antipsychotic | 1140867444 | Risperidone |  |  |  |  |  |
|  | Antipsychotic | 1140867420 | Clozapine |  |  |  |  |  |
|  | Antipsychotic | 1140867414 | Loxapac 10mg capsule |  |  |  |  |  |
|  | Antipsychotic | 1140867406 | Loxapine |  |  |  |  |  |
|  | Antipsychotic | 1140867398 | Fluphenazine decanoate |  |  |  |  |  |
|  | Antipsychotic | 1140867332 | Trifluperidol |  |  |  |  |  |
|  | Antipsychotic | 1140867304 | Sulpiride |  |  |  |  |  |
|  | Antipsychotic | 1140867218 | Pimozide |  |  |  |  |  |
|  | Antipsychotic | 1140867208 | Perphenazine |  |  |  |  |  |
|  | Antipsychotic | 1140867184 | Haldol 5mg tablet |  |  |  |  |  |
|  | Antipsychotic | 1140867168 | Haloperidol |  |  |  |  |  |
|  | Antipsychotic | 1140867156 | Moditen 1mg tablet |  |  |  |  |  |
|  | Antipsychotic | 1140867150 | Flupenthixol |  |  |  |  |  |
|  | Antipsychotic | 1140867136 | Neulactil 2.5mg tablet |  |  |  |  |  |
|  | Antipsychotic | 1140867134 | Pericyazine |  |  |  |  |  |
|  | Antipsychotic | 1140867122 | Nozinan 25mg tablet |  |  |  |  |  |
|  | Antipsychotic | 1140867084 | Droperidol |  |  |  |  |  |
|  | Antipsychotic | 1140867078 | Benperidol |  |  |  |  |  |
|  | Antipsychotic | 1140863416 | Largactil 10mg tablet |  |  |  |  |  |
|  | Antipsychotic | 1140856052 | Chlorprothixene |  |  |  |  |  |
|  | Antipsychotic | 1140856004 | Moditen enanthate 25mg/ml injection |  |  |  |  |  |
|  | Antipsychotic | 1140909800 | Flupentixol |  |  |  |  |  |
|  | Antipsychotic | 1140909802 | Levomepromazine |  |  |  |  |  |
|  | Antipsychotic | 1140909804 | Pipotiazine |  |  |  |  |  |
|  | Antipsychotic | 1140928916 | Olanzapine |  |  |  |  |  |
|  | Antipsychotic | 1141152848 | Quetiapine |  |  |  |  |  |
|  | Antipsychotic | 1141153490 | Amisulpride |  |  |  |  |  |
|  | Antipsychotic | 1141177762 | Risperdal 0.5mg tablet |  |  |  |  |  |
|  | Antipsychotic | 1141195974 | Aripiprazole |  |  |  |  |  |
|  | Antipsychotic | 1141202024 | Abilify 5mg tablet |  |  |  |  |  |
|  | Antipsychotic | 1140882382 | Butyrophenone product |  |  |  |  |  |
|  | Antipsychotic | 1140879750 | Thioridazine |  |  |  |  |  |
|  | Antipsychotic | 1140879746 | Promazine |  |  |  |  |  |
|  | Antipsychotic | 1140879674 | Pipothiazine |  |  |  |  |  |
|  | Antipsychotic | 1140879658 | Chlorpromazine |  |  |  |  |  |
|  | Antipsychotic | 1140882100 | Zuclopenthixol |  |  |  |  |  |
|  | Antipsychotic | 1140882098 | Fluphenazine |  |  |  |  |  |
|  | Antipsychotic | 1140882320 | Clozaril 25mg tablet |  |  |  |  |  |
|  | Antipsychotic | 1141169714 | Zotepine |  |  |  |  |  |
|  | Anxiety | 1140872236 | Neurontin 100mg capsule | 2998 | 224237 | 9 | 929 | Chi2[1]=0.674, p=0.412 |
|  | Anxiety | 1140872228 | Gabapentin |  |  |  |  |  |
|  | Anxiety | 1140872150 | Clonazepam |  |  |  |  |  |
|  | Anxiety | 1140863442 | Oxazepam |  |  |  |  |  |
|  | Anxiety | 1140863374 | Nobrium 5mg capsule |  |  |  |  |  |
|  | Anxiety | 1140863364 | Ativan 1mg tablet |  |  |  |  |  |
|  | Anxiety | 1140863308 | Alprazolam |  |  |  |  |  |
|  | Anxiety | 1140863302 | Lorazepam |  |  |  |  |  |
|  | Anxiety | 1140863268 | Clobazam |  |  |  |  |  |
|  | Anxiety | 1140863262 | Chlormezanone |  |  |  |  |  |
|  | Anxiety | 1140863250 | Valium 2mg/5ml syrup |  |  |  |  |  |
|  | Anxiety | 1140863244 | Valium 2mg tablet |  |  |  |  |  |
|  | Anxiety | 1140863210 | Normison 10mg capsule |  |  |  |  |  |
|  | Anxiety | 1140863202 | Temazepam |  |  |  |  |  |
|  | Anxiety | 1140863194 | Mogadon 5mg tablet |  |  |  |  |  |
|  | Anxiety | 1140863182 | Nitrazepam |  |  |  |  |  |
|  | Anxiety | 1140863176 | Lormetazepam |  |  |  |  |  |
|  | Anxiety | 1140863152 | Diazepam |  |  |  |  |  |
|  | Anxiety | 1140863120 | Loprazolam |  |  |  |  |  |
|  | Anxiety | 1140863110 | Flurazepam |  |  |  |  |  |
|  | Anxiety | 1140863022 | Noctec 500mg capsule |  |  |  |  |  |
|  | Anxiety | 1140855914 | Triazolam |  |  |  |  |  |
|  | Anxiety | 1140855856 | Valium 10mg suppository |  |  |  |  |  |
|  | Anxiety | 1141157496 | Diazepam product |  |  |  |  |  |
|  | Anxiety | 1141168436 | Tiagabine |  |  |  |  |  |
|  | Anxiety | 1141200004 | Pregabalin |  |  |  |  |  |
|  | Mixed antidepressant antipsychotic | 1140867948 | Amitriptyline hydrochloride+perphenazine 10mg/2mg tablet | 15 | 227220 | 0 | 938 | >0.999 |
|  | Mixed antidepressant antipsychotic | 1140867944 | Tranylcypromine+trifluoperazine 10mg/1mg tablet |  |  |  |  |  |
|  | Mixed antidepressant antipsychotic | 1140867942 | Fluphenazine hcl+nortriptyline 500micrograms/10mg tablet |  |  |  |  |  |
|  | Mixed antidepressant antipsychotic | 1140867940 | Fluphenazine hydrochloride+nortriptyline 1.5mg/30mg tablet |  |  |  |  |  |
|  | Mixed antidepressant antipsychotic | 1140867930 | Motival tablet |  |  |  |  |  |
|  | Mixed antidepressant antipsychotic | 1140882240 | Motipress tablet |  |  |  |  |  |
|  | Mood Stabiliser | 1140872302 | Lamictal 25mg tablet | 1658 | 225577 | 4 | 934 | Chi2[1]=0.805, p=0.370 |
|  | Mood Stabiliser | 1140872290 | Lamotrigine |  |  |  |  |  |
|  | Mood Stabiliser | 1140872214 | Valproic acid |  |  |  |  |  |
|  | Mood Stabiliser | 1140872200 | Epilim 100mg crushable tablet |  |  |  |  |  |
|  | Mood Stabiliser | 1140872198 | Sodium valproate |  |  |  |  |  |
|  | Mood Stabiliser | 1140872072 | Tegretol 100mg tablet |  |  |  |  |  |
|  | Mood Stabiliser | 1140872064 | Carbamazepine product |  |  |  |  |  |
|  | Mood Stabiliser | 1140867520 | Li-liquid 5.4mmol/5ml oral solution |  |  |  |  |  |
|  | Mood Stabiliser | 1140867518 | Litarex 564mg m/r tablet |  |  |  |  |  |
|  | Mood Stabiliser | 1140867504 | Priadel 200mg m/r tablet |  |  |  |  |  |
|  | Mood Stabiliser | 1140867498 | Liskonum 450mg m/r tablet |  |  |  |  |  |
|  | Mood Stabiliser | 1140867494 | Camcolit 250 tablet |  |  |  |  |  |
|  | Mood Stabiliser | 1140867490 | Lithium product |  |  |  |  |  |
|  | Mood Stabiliser | 1140910976 | Lithonate 400mg m/r tablet |  |  |  |  |  |
|  | Mood Stabiliser | 1140917270 | Li-liquid 509mg/5ml oral solution |  |  |  |  |  |
|  | Mood Stabiliser | 1140923484 | Topiramate |  |  |  |  |  |
|  | Mood Stabiliser | 1141172838 | Depakote 250mg e/c tablet |  |  |  |  |  |
|  | Mood Stabiliser | 2038459704 | Carbamazepine |  |  |  |  |  |
|  | Side Effect | 1140865394 | Hyoscine butylbromide | 213 | 227022 | 0 | 938 | >0.999 |
|  | Side Effect | 1140883476 | Procyclidine |  |  |  |  |  |
|  | Side Effect | 1140883510 | Benzhexol |  |  |  |  |  |
|  | Side Effect | 1140883514 | Benztropine |  |  |  |  |  |
|  | Side Effect | 1140909816 | Trihexyphenidyl |  |  |  |  |  |
|  | Side Effect | 1140909818 | Benzatropine |  |  |  |  |  |
|  | Side Effect | 1141157482 | Hyoscine product |  |  |  |  |  |
|  | Side Effect | 1140882232 | Kwells tablet |  |  |  |  |  |
|  | Side Effect | 1140882104 | Hyoscine |  |  |  |  |  |
